# Ethylene Oxide Exposure in U.S. Populations Residing Near Sterilization and Other Industrial Facilities: Context Based on Endogenous and Total Equivalent Concentration Exposures

**DOI:** 10.3390/ijerph18020607

**Published:** 2021-01-12

**Authors:** Patrick J. Sheehan, Ryan C. Lewis, Christopher R. Kirman, Heather N. Watson, Eric D. Winegar, James S. Bus

**Affiliations:** 1 Health Sciences, Exponent, Inc., Oakland, CA 94612, USA; rlewis@exponent.com (R.C.L.); ewinegar@exponent.com (E.D.W.); 2 Summit Toxicology, Bozeman, MT 59722, USA; ckirman@summittoxicology.com; 3 Data Sciences, Exponent, Inc., Menlo Park, CA 94025, USA; hwatson@exponent.com; 4 Health Sciences, Exponent, Inc., Alexandria, VA 22314, USA; jbus@exponent.com

**Keywords:** ethylene oxide, exposure metrics, endogenous equivalent concentration, total equivalent concentration, exposure contextualization, exposure science

## Abstract

Given ubiquitous human exposure to ethylene oxide (EO), regardless of occupation or geography, the current risk-specific concentrations (RSCs: 0.0001–0.01 ppb) from the U.S. Environmental Protection Agency (EPA) cancer risk assessment for EO are not useful metrics for managing EO exposures to the general U.S. population. The magnitude of the RSCs for EO are so low, relative to typical endogenous equivalent metabolic concentrations (1.1–5.5 ppb) that contribute ~93% of total exposure, that the RSCs provide little utility in identifying excess environmental exposures that might increase cancer risk. EO monitoring data collected in the vicinity of eight EO-emitting facilities and corresponding background locations were used to characterize potential excess exogenous concentrations. Both 50th and 90th percentile exogenous exposure concentrations were combined with the 50th percentile endogenous exposure concentration for the nonsmoking population, and then compared to percentiles of total equivalent concentration for this population. No potential total exposure concentration for these local populations exceeded the normal total equivalent concentration 95th percentile, indicating that excess facility-related exposures are unlikely to require additional management to protect public health.

## 1. Introduction

In 2016, the U.S. Environmental Protection Agency (EPA) Integrated Risk Information System (IRIS) assessment of ethylene oxide (EO) estimated risk-specific concentrations (RSCs) of 0.0001 to 0.01 parts per billion by volume (ppb), associated with a 10^−6^ to 10^−4^ increase in inhalation cancer risk, respectively [1]. As everyone is exposed to single-digit, parts per billion (ppb) equivalent levels of endogenous, metabolically-produced EO and fractions of ppb levels from inhalation of ambient air, regardless of their occupation or location of residence [2,3,4,5], these exceedingly low RSCs relative to endogenous levels and variability raise questions as to their practical utility in risk management of public exogenous EO exposure, as well as their scientific merit. In contrast, the Texas Commission on Environmental Quality (TCEQ) using the same cohort data as EPA IRIS, but a different dose-response model, estimated 10^−6^ to 10^−4^ RSCs of 0.24 to 24 ppb, respectively [6]. We note that neither the EPA nor TCEQ addressed the assumption of low exposure concentrations for early sterilization workers, which was shown to be invalid [7]. The EPA assessment short comings left a gap in our ability to interpret the health significance of general population exogenous EO background exposures from non-industrial and natural EO sources, and, perhaps more importantly, local general population exposures from point source industrial emissions. Kirman and Hays [2] and Kirman et al. [3] proposed an endogenous exposure metric, endogenous equivalent concentration, to provide context to exogenous exposures for decision-making by risk managers. This study proposes and evaluates the utility of a total exposure metric (total equivalent concentration) incorporating both background endogenous and exogenous EO exposures to help inform the health significance of excess ambient air exposures to EO.

As background, the primary limitation with the EPA RSCs for EO is that they are so low relative to the amount and variability in typical background endogenous and exogenous general population exposures that they have limited utility of distinguishing a health-significant increase in environmental exposure for risk managers. In addition, these RSCs are below the capabilities of current technology to measure these EO concentrations in ambient air (current method limit of detection (LOD) ~0.025–0.040 ppb). These RSCs are also more than two orders of magnitude below the EO ambient air concentrations, which are not associated with industrial emission sources (mean of 0.13 ppb based on recent EPA monitoring [5]). More importantly, these RSCs are more than three orders of magnitude below airborne concentrations equivalent to endogenously produced EO, in the general nonsmoking population (mean concentrations of ~1.9 and 2.9 ppb) based on data evaluated by Kirman and Hays [2], based on data from published unexposed control subjects [8,9,10,11,12,13,14,15,16,17] and Kirman et al. [3], and based on data from nonsmoking U.S. individuals [4], respectively. For additional perspective, as described by Bogen et al. [7], these RSCs are up to seven orders of magnitude below the levels of EO to which 1930s–1970s sterilization operators and other highly exposed sterilization workers in the National Institute for Occupational Safety and Health (NIOSH) cohort used by EPA in its risk assessment were exposed (50,000 to >100,000 ppb). Based on these comparisons, it is obvious that the EPA RSCs are incapable of providing useful benchmarks to evaluate the health significance of such low general population EO exposures. In addition, regulatory use of these RSCs inadvertently led to substantial confusion on the health significance of exogenous EO exposures among emitting facility management and locally exposed populations, resulting in public fear and closure of key medical supply sterilization operations [18,19], during the COVID-19 pandemic.

Total EO exposures in humans were characterized using hemoglobin adducts, specifically 2-hydroxyethylvaline (HEV), which serves as a useful biomarker of exposure, regardless of the exposure pathway. A summary of HEV levels in non-smokers and smoker populations and the relatively high variability in adduct levels in these population is provided in Kirman and Hays [2] and Kirman et al. [3]. The distribution of HEV levels for the non-smoking general population, therefore, describes the expected range of adducts from background exposure from endogenous and exogenous sources to which everyone is exposed in their daily lives. Kirman et al. [3] describe the three primary pathways of EO exposure to the non-occupational general population in the U.S.—(1) EO is produced endogenously in the body via multiple pathways, including ethylene from bacterial pathways in the gastrointestinal lumen and systemic enzymatic production from methionine and 2-keto-4-methylthiobutyric acid, (2) exogenous ethylene inhaled from ambient air and converted metabolically to EO, and (3) exogenous EO inhaled from ambient air from sources other than industrial emissions. To compare the contribution of these sources to total background EO exposure, HEV levels were converted to endogenous equivalent air concentrations (i.e., the amounts of exogenous EO exposures necessary to result in the measured endogenous HEV adduct concentrations, regardless of the three potential sources). Based on data collected between 1986 and 2012, a mean and standard deviation (SD) endogenous equivalent concentration were calculated (1.9 ± 1.3 ppb) [2]. More recently, Kirman et al. [3] recalculated equivalent levels incorporating two important changes—(1) HEV values for non-smokers in the U.S. population between 2013 and 2016 were used as the basis, instead of relying upon pooled data for unexposed experimental control subjects in the previous analysis, and (2) background levels of HEV were adjusted for contributions from exogenous exposure pathways (via ambient air), instead of assuming that the contributions from exogenous exposures were negligible. The new endogenous equivalent mean was higher than that previously calculated (2.9 ± 2.3 ppb).

Thus, the endogenous pathway is a major source of the total EO exposure among the general population, however, everyone also receives a small exogenous exposure from ambient air. As described by Kirman et al. [3], the specific contributions of all sources to the mean total EO exposure concentration, as measured by the HEV adducts among the non-smoking general population (2.9 ppb; background EO exposure), were estimated as follows: (1) endogenous metabolism, 2.7 ppb (~93.1%); (2) exogenous ethylene inhalation and metabolism, 0.04 ppb (~1.4%); and (3) exogenous EO inhalation, 0.16 ppb (~5.5%). The contribution from ethylene metabolism from endogenous and exogenous sources (~94.5%) clearly indicates that exogenous EO exposure is a minor contributor to total background exposure among individuals in the non-smoking general population. As such, it is reasonable to conclude that EO exposure concentrations among the non-smoking local population near emitting facilities could be compared to the general population endogenous equivalent concentrations (based only on the endogenous contribution) [2,3] or perhaps more representatively, total equivalent concentrations (background endogenous and exogenous EO contributions), as exposure metrics to provide context for EO risk management. An example of this approach was shown in Kirman et al. [3]. This practical alternative risk management approach is based on the premise that increased exogenous exposure does not necessarily pose a significant increase in the risk of disease if the total exposure does not exceed the upper bound of the non-smoking total background exposure concentration range. This approach is equivalent to the medical interpretation of risk, based on the comparison of the level of a clinical measure to the normal population range (i.e., if the measured clinical value of interest is in the normal population range, such a value is not interpreted as an indicator of a treatment-required disease state).

The goal of this paper is to assess the utility of the normal nonsmoking population total equivalent exposure range to provide context for the EO exposures for local populations residing in the vicinity of sterilization and other EO emitting industrial facilities. To evaluate the utility of this exposure metric to inform risk management decisions, this study included the following:

- Development of distribution of percentiles of the nonsmoking general population endogenous equivalent concentrations and total equivalent concentrations.
- Characterization of recently measured ambient EO concentrations in the vicinity of sterilization and other EO emitting facilities and at corresponding representative background sites to assess exogenous exposure concentration above background levels.
- Assessment of the relative importance of excess EO concentrations in the vicinity of emitting facilities by comparing a central tendency or upper bound ambient air EO concentration added to the general population 50th percentile endogenous equivalent concentration, measures of total exposure, to the normal percentile range of nonsmoking general population total equivalent concentrations.

## 2. Materials and Methods

This section identifies the data and method used to characterize endogenous equivalent and total equivalent exposure concentrations, and EO concentrations in ambient air in the vicinity of emitting facilities and associated background locations.

### 2.1. Equivalent Exposure Concentrations

Recently, the Centers for Disease Control and Prevention (CDC) released biomonitoring data for HEV concentrations in the general U.S. population collected over two sampling periods (2013–14, 2015–16) for non-smokers (3841 persons) and smokers (93 persons) [4]. These data, which reflect a larger and more diverse population than assessed in Kirman and Hays [2], demonstrate that there are differences in HEV levels (and therefore in the total ethylene oxide exposure), depending on smoking status, age, and gender. These HEV levels in human blood can be converted to endogenous equivalent levels of ethylene oxide, based on the following equation between HEV adduct levels and measured occupational exposures to ethylene oxide, adjusted to general population exposures [2].

(1) $$\mathrm{HEV}\;\left(\mathrm{pmol}/g\;\mathrm{Hb}\right)\;=\;10.9\;*\;\left[\mathrm{EO},\;\mathrm{ppb}\;\mathrm{continuous}\right]$$

Endogenous equivalent levels reflect air concentrations of ethylene oxide that are equivalent to the levels that are produced endogenously. The endogenous equivalent levels are calculated here for ethylene oxide, based on (1) HEV values from CDC for non-smokers in the U.S. [4], and (2) background levels of HEV adjusted for contributions from exogenous EO exposure pathway (via ambient air [5]). This process produces two exposure metrics—(1) endogenous equivalent concentrations that represent continuous exposure levels produced metabolically from endogenous ethylene, and (2) total equivalent concentrations that represent continuous exposure produced both metabolically and from inhaled ambient air. Distributions of these metrics were developed to describe the percentile concentrations of non-smoking general population normal exposure.

### 2.2. EO Concentrations in Ambient Air Near Emitting U.S. Facilities and Associated Background Locations

Ambient air samples were collected at selected locations, in the vicinity of emitting U.S. facilities, to characterize EO concentrations for local populations. To date (as of 19 October 2020), we are aware of publicly available data concerning monitoring programs associated with seven sterilization facilities (Medline, Sterigenics-Illinois, Sterigenics-Georgia, Becton-Dickinson, Sterilization Services, Terumo, and Viant) and one specialty chemical facility (Vantage). With each monitoring programs, samples were also collected at various distances from the facility to characterize the reference ambient air EO concentrations from non-industrial sources (background concentrations), which, in turn, would help inform what fraction of the EO in air in the vicinity of a facility might be related to facility emissions.

#### 2.2.1. Available Data

The EO monitoring programs for the eight facilities providing data that were evaluated in this manuscript are described in Table 1; the raw data can be found in Supplementary Material S1. All samples were long-term (24 h and, to a much lesser extent, 12 h) and were collected at multiple locations, generally within 2000 m of the facility and over multiple days per month and months per year. None of the monitoring represented samples collected over an entire year, although several programs likely captured most seasonal differences in wind direction. The one exception was the Viant facility for which there was very limited temporal data collected. Samples at the Medline and Terumo facilities were unique because they were collected before and after installation of additional EO emissions control technology. Facility monitoring programs collected local background/reference samples at locations between 2000 and 10,000 m from the facility location. Again, the exception was Viant, where no reference samples were identified, so four location >2100 m from the facility were selected as the background reference locations. In addition, the background locations for the three Georgia facilities (Coffee Park and South DeKalb, Atlanta, GA, USA) were more state than local background locations and were located at substantial distances from these facilities (>22,500 to >45,000 m for the closest background location).

Additionally, there were national ambient EO monitoring data collected under the EPA National Air Toxics Trends Stations (NATTS) and Urban Air Toxics (UAT) monitoring Programs. The goals of these monitoring program were to identify and reduce air toxics of greatest potential concern, for contribution to the general population risk.

#### 2.2.2. EO Collection and Analytical Methods Applied to EO Monitoring

Concentrations of EO from national monitoring program samples and facility monitoring samples were determined by the protocols in EPA Compendium Method TO-15 and its recent update TO-15A, both of which were incorporated into the NATTS Technical Assistance Document (TAD) [30]. The NATTS method used Method TO-15 as its foundation—(1) sample collection of ambient air usually for a 24-h period into an evacuated summa or silica-coated canister; (2) pre-concentration of an aliquot to remove permanent gases and carbon dioxide; (3) moisture management techniques to remove interfering water; (4) injection of the sample into a gas chromatograph for separation of individual species; and (5) detection by mass spectrometry, either by full scan or (mostly) the selected ion mode. The current method was based on refinements of the NATTS TAD specifications undertaken throughout the period in which these samples were collected. Sensitivity was determined by EPA’s contract laboratory on three separate systems, using 7 spiked canisters at 0.1 ppb (by volume), resulting in method detection limits from 0.025 to 0.061 ppb [31].

#### 2.2.3. Statistical Methods

Non-detect data were assigned a value of limit of detection divided by 2, and the collocated samples were averaged and treated as a single sample in subsequent analyses. Exploratory analyses were performed, examining not only the EO concentration by location over time, but also the distribution by facility and location, to investigate skewness and potential outliers. Two unusually high concentration samples were removed from the Georgia background data set, but no potential outliers were removed from other background data sets or any facility area data sets. The EO concentration of each sub-group was tested for normality or lognormality. For each facility and location, the mean and SD were calculated, along with the 50th and 90th percentiles, based on the lognormal distribution.

### 2.3. Comparing EO Exposure Concentrations to Endogenous Equivalent Concentration Benchmarks

Three exposure comparisons were proposed to provide context for interpreting the health implication of exposure concentrations near emitting facilities—(1) compare the facility mean concentration to local background mean concentration (i.e., increase above background), (2) compare the facility mean concentration to the endogenous equivalent mean concentration (i.e., fraction of endogenous equivalent), and (3) add the facility 50th and 90th percentile concentration to the 50th percentile endogenous equivalent concentration, and compare this total exposure metric to the non-smoking population percentile total equivalent concentration.

## 3. Results

### 3.1. Total Equivalent and Endogenous Equivalent Concentrations

Kirman and Hays [2] initially proposed endogenous equivalent concentrations in air for EO (termed action levels), considering it endogenous HEV production. These endogenous equivalent concentrations were recalculated for EO, based on the mean HEV values from CDC for nonsmokers in the U.S. [4] and levels of HEV were adjusted for contributions from exogenous exposure pathways, instead of assuming that contributions from exogenous exposures were negligible [3]. Table 2 summarizes the percentiles of the distribution of the nonsmoking U.S. population for the total equivalent concentration metric, the focus of this evaluation, as well as the endogenous equivalent concentration metric. The total equivalent concentrations were about 0.2 ppb greater than the endogenous concentrations, as exogenous background concentrations constitute a small fraction of total exposure.

### 3.2. Facility Vicinity and Background EO Concentrations

**Figure 1:**
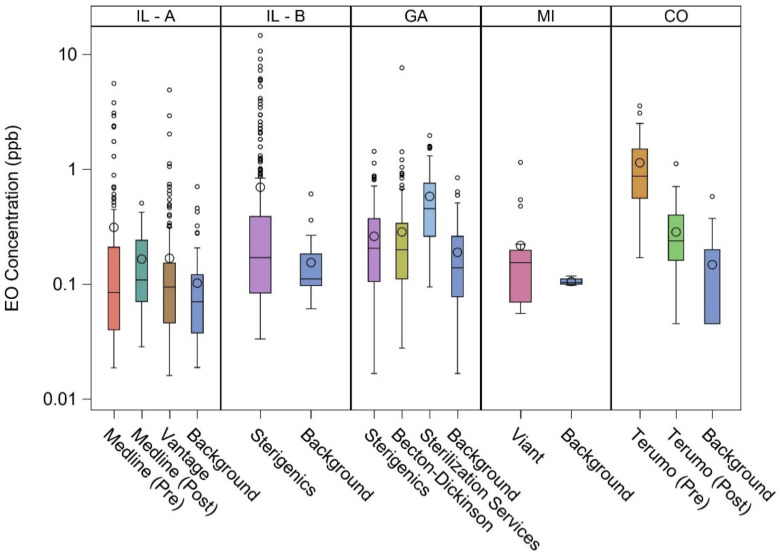
Distribution of ambient air EO concentrations in the vicinity of eight EO-emitting industrial facilities and at corresponding representative background locations. Length of box = interquartile range (IQR or P75–P25); horizontal line = P50; large circle = mean; lower whisker = P25 – (1.5 × IQR); upper whisker = P75 + (1.5 × IQR); small circle = potential outlier.

Figure 1 shows the distribution of EO concentrations in the vicinity of the eight facilities and at corresponding representative background locations. It is clear that the concentrations were positively skewed, especially near these facilities, indicating that the median was a more representative measure of central tendency than the mean. Except for Terumo (pre-additional EO emission controls), median concentrations in the vicinity of the facilities were less than 0.5 ppb. Peak EO concentrations at Terumo and Medline were greatly reduced with implementation of additional emission controls.

As shown in Table 3, these EO concentrations were separately fit to the lognormal distributions, from which the selected percentiles were estimated. For facility vicinity EO concentrations, Vantage had the lowest 50th percentile (0.09 ppb) and 90th percentile (0.43 ppb), whereas Terumo (pre-additional emission controls) had the highest 50th percentile (0.87 ppb) and 90th percentile (3.1 ppb); the majority had a 50th percentile less than about 0.20 ppb and a 90th percentile less than about 0.90 ppb. The 50th percentile (0.07 to 0.13 ppb) and 90th percentile (0.12 to 0.56 ppb) for the background locations were comparatively less variable. These metrics provide further support for characterizing ambient air EO distributions as positively skewed.

### 3.3. National Background and Local Background EO Concentrations

Background EO concentrations in ambient air represent EO emissions from natural and anthropogenic sources, excluding point source industrial emissions. Background sources include EO released from microbial processes in soil and exhaust from automobiles and stationary sources of hydrocarbon combustion [32]. An overall mean background EO concentration in ambient air was nationally (0.13 ppb) calculated for EO monitoring at 27 monitoring locations, as part of the EPA’s NATTS and UAT monitoring program stations, sampled between October 2018 and September 2019 [5]. The overall mean for EO concentration for local background EO concentration (0.14 ppb) for the eight industrial facilities evaluated herein was calculated for 27 individual locations sampled at various times between September 2018 and August 2020 (Table 4). The national and local background monitoring programs thus contained the same number of monitoring locations, were sampled in overlapping time-periods and provided equivalent results.

**Figure 2:**
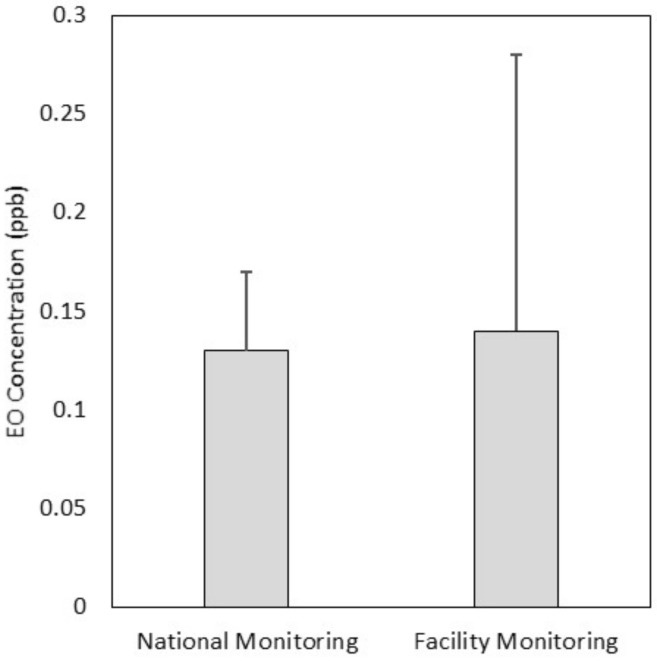
Overall mean (top of box) and SD (whisker) of ambient air EO concentrations across 27 national monitoring locations and across the 27 individual locations aggregated into five background locations for the facilities characterized in this manuscript.

As shown in Figure 2, the national ambient air monitoring sites mean background EO concentration and the local facility-related monitoring sites mean background EO concentration were comparable (0.13–0.14 ppb); a Wilcoxon statistical comparison of these means showed no statistically significant difference. The overall national mean calculated here (0.13 ppb) was slightly lower than the mean calculated from an earlier data set (0.16 ppb) [3].

### 3.4. Risk Management Context for Near Facility Potential Population Exposure Concentrations

**Figure 3:**
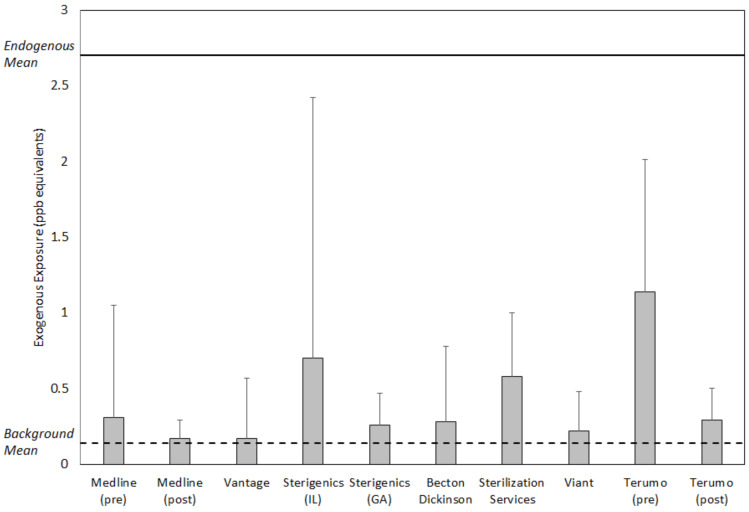
Mean (top of box) and SD (whisker) of ambient air EO concentrations in the vicinity of eight sterilization and other EO-emitting industrial facilities, relative to overall facility-specific background mean EO concentration (0.14 ppb) and endogenous mean EO concentration for the non-smoking U.S. population (2.7 ppb).

**Figure 4:**
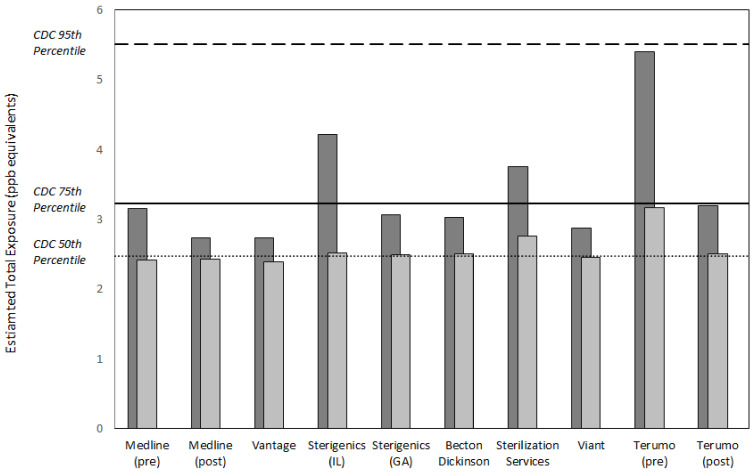
Estimated total equivalent exposure to EO in the vicinity of eight sterilization and other EO-emitting industrial facilities relative to that of the 50th, 75th, and 95th percentiles of the non-smoking U.S. population (2.5, 3.2, and 5.5 ppb, respectively). Light gray = [50th percentile endogenous equivalent for the non-smoking U.S. population, or 2.3 ppb] + [50th percentile EO concentration for the facility area from Table 3]. Dark gray = same as light gray, except using 90th percentile EO concentration for the facility area from Table 3.

Figure 3 and Figure 4 summarize the values for exposure metrics that provide context for interpreting the health significance of potential EO exposure concentrations in the vicinity of the eight emitting facilities evaluated in this study. In all cases, the mean concentration in the vicinity of the facility was no greater than eight times the mean background concentration for the facility and in a substantial majority of the cases, was greater by two or less (Figure 3). Thus, the facility mean concentrations were not substantially elevated above the related background mean concentrations. Similarly, the facility mean concentration composed a small fraction of the endogenous equivalent mean concentration, generally 0.1 or less. This finding was consistent with previously reported analyses that exogenous concentrations constitute a small fraction of endogenous EO exposure concentrations [3]. More importantly, the final two metrics that evaluate the significance of adding the exogenous facility area 50th and 90th percentile EO concentration to nonsmoking population 50th percentile endogenous concentration, showed that these total exposure concentrations were within the normal population range of total equivalent exposure concentrations experienced by nonsmoking populations in the United States (Figure 4). For the 50th percentile exogenous addition, all total concentration values were within the 75th percentile total equivalent concentration and the vast majority were approximately equal to 50th percentile total equivalent concentration. For the 90th percentile addition, all total concentration values were within the 95th percentile total equivalent concentration and the vast majority were equal to or less than the 75th percentile total equivalent concentration.

## 4. Discussion

The limitations in the utility of the EPA IRIS cancer risk assessment for EO left a serious gap in confidence in interpreting the health significance of general population EO exposure, and of particular interest, managing exposure risk for local populations in the vicinity of EO emitting sterilization and other industrial facilities. As everyone is exposed to EO and endogenous exposure from ethylene metabolism is the predominant pathway, Kirman and Hays [2] and Kirman et al. [3] calculated endogenous equivalent concentration values for nonsmokers to help risk managers in search of a pragmatic science-based approach to informing management potential EO risk for exposed general populations. The evaluations herein provide an additional nonsmoker population exposure metric, total equivalent concentration, focused specifically on providing context to the health significance of EO exposures for populations in the vicinity of sterilization facilities and other industrial emitting facilities, as these population receive an additional exogenous EO exposure beyond the general population background exposure. Both the endogenous equivalent concentration and total equivalent concentration metrics are based on the premise that exposure concentrations within the normal ranges of these metrics should not significantly increase risk. This premise is consistent with clinical metrics for which the risk of disease does not increase significantly until the values are above the healthy population normal range defined by individual variability within the population.

The health significance of facility area concentrations in this analysis was defined as a continuous total exposure concentration (50th percentile endogenous concentration + facility area exogenous concentration) greater than the 95th percentile total equivalent EO concentration, a statistical upper bound of the normal nonsmoking population range. The analyses summarized in Table 4 above show that the facility mean concentrations were not substantially above the representative mean background concentrations (frequently <2 fold) and compose a small fraction of the mean endogenous equivalent concentration (generally <0.1). More importantly, this analysis showed that the excess exogenous concentrations experienced near facilities did not contribute substantially to the nonsmoking population total EO exposure. This was indicated by the total equivalent concentration percentile comparisons, which showed that all total concentration values were within the 95th percentile total equivalent concentration for background populations not exposed to industry emission sources, and the vast majority were equal to or less than the 75th percentile total equivalent concentration.

Kirman et al. [3] also evaluated exogenous EO exposures from populations of smoking individuals as well as non-smoking individuals. Since increased prevalence of EO-associated cancer (lymphoid and breast) was not observed with the elevated exposures to EO from smoking where endogenous production levels were up to an order of magnitude higher than for non-smokers [33], endogenous equivalent and total equivalent concentrations for nonsmokers in the general population should be considered to be conservative metrics in informing risk management decisions for locally exposed populations.

The endogenous equivalent and total equivalent concentration risk management metrics are believed to be health conservative, supported by current HEV data for U.S. populations [4], and the science of EO metabolic production is easy to interpret. Thus, these should provide additional risk management tools to contextualize general population exogenous EO exposures during this period of uncertainty in EO cancer risk assessment. An additional benefit of the HEV data is that they can be informative for contextualizing individual adduct measures from populations residing near EO emitting facilities.

## 5. Conclusions

Total equivalent concentration, derived from adduct data for nonsmokers in the U.S., provides an exposure metric to inform risk management decisions for individuals and populations in the vicinity of emitting industrial facilities, accounting for their total EO exposure. Without useful RSCs, this total exposure metric can provide important context as to whether the increased general population EO exposures resulting from industrial emissions are sufficient to pose an increased risk of cancer.

## Figures and Tables

**Table 1:** Summary of publicly-available datasets concerning ambient EO concentrations in the vicinity of seven sterilization and one other EO-emitting industrial facility ^1^ and at corresponding representative background locations.

Facility	Location	Year	Season ^1^	*n* (sites) ^2,3^	Dist. ^4^	Dur. ^5^
Medline ^6^ [20]	Waukegan, IL	19/20	A, B, C	174 (5)	~190–1660	24
Vantage [20]	Gurnee, IL	19/20	A, B, C	250 (5)	~260–1130	24
Background [20]	Gurnee, IL	19/20	A, B, C, D	98 (2)	>2440	24
Sterigenics [21]	Willowbrook, IL	18/19	C, D	237 (8)	~100–1700	24
Background [22]	Chicago, IL area	18/19	C	28 (14)	>9600	12
Sterigenics [23]	Smyrna, GA	19/20	A, B, C, D	211 (7)	~280–1970	24
BD ^7^ [24]	Covington, GA	19/20	A, B, C, D	267 (8)	~60–1980	24
SS ^8^ [25]	Atlanta, GA	19/20	A, B, D	64 (3)	~150–840	24
Background [26]	General Coffee, GA	19/20	A, B, C, D	25 (1)	>290,000	24
Background [27]	South DeKalb, GA	19/20	A, B, C, D	58 (2)	>22,500	24
Viant [28]	Grand Rapids, MI	18/19	A, B, C, D	20 (12)	~80–1520	24
Background [28]	Grand Rapids, MI	19	A	4 (4)	>2100	24
Terumo [29]	Lakewood, CO	18	B, C	84 (8)	~40–1570	24
Background [29]	Denver, CO area	18	C	18 (4)	>4500	24

^1^ Season in which samples were collected: A, spring (Mar.–May); B, summer (Jun.–Aug.); C, fall (Sept.–Nov.); D, winter (Dec.–Feb.); ^2^ total number of samples (total number of sampling sites); ^3^ excludes facility data during temporary shutdown or after permanent shutdown; ^4^ distance of sample sites from facility in meters; ^5^ duration of samples in hours; ^6^ did not include measurement data during which the facility was temporarily shut down; ^7^ BD, Becton Dickinson; and ^8^ SSG, Sterilization Services.

**Table 2:** Total equivalent and endogenous equivalent EO air concentrations (ppb) using HEV levels (pmol/g Hb) in percentiles of nonsmoker population in the United States, as originally reported by Kirman et al. [3], based on CDC [4] data.

Percentile	HEV_unadj_^1^	Total Eq. ^2^	HEV_adj_^3^	Endog. Eq. ^4^
	pmol/g Hb	ppb	pmol/g Hb	ppb
P5	13.4	1.3	11.2	1.0
P10	16.0	1.5	13.8	1.3
P25	20.8	1.9	18.6	1.7
P50	27.0	2.5	24.8	2.3
P75	35.1	3.2	32.9	3.0
P90	47.5	4.4	45.3	4.2
P95	60.1	5.5	57.9	5.3

^1^ HEV_unadj_, HEV level from exogenous + endogenous sources; ^2^ total equivalent EO concentration from endogenous and background exogenous source; ^3^ HEV_adj_, HEV level from endogenous sources only; and ^4^ endogenous equivalent EO concentration from individual metabolism.

**Table 3:** Statistical characterization of EO concentrations (ppb) in ambient air in the vicinity of eight EO-emitting industrial facilities and at corresponding representative background locations.

Facility (Controls ^1^)	Mean ± SD	P50 ^2^	P90 ^3^
Medline (pre)	0.31 ± 0.74	0.11	0.86
Medline (post)	0.17 ± 0.12	0.13	0.43
Vantage	0.17 ± 0.40	0.09	0.43
Background	0.10 ± 0.11	0.07	0.26
Sterigenics ^4^	0.70 ± 1.72	0.22	1.92
Background	0.15 ± 0.11	0.13	0.31
Sterigenics ^5^	0.26 ± 0.21	0.19	0.77
Becton Dickinson	0.28 ± 0.50	0.20	0.73
Sterilization Services	0.58 ± 0.42	0.46	1.45
Background	0.19 ± 0.16	0.13	0.56
Viant	0.22 ± 0.26	0.15	0.57
Background	0.11 ± 0.01	0.11	0.12
Terumo (pre)	1.14 ± 0.87	0.87	3.10
Terumo (post)	0.29 ± 0.21	0.21	0.89
*Background*	0.15 ± 0.15	0.10	0.44

^1^ Pre- or post-installation of additional EO emissions controls; ^2^ P50, lognormal estimate of the 50th percentile; ^3^ P90, lognormal estimate of the 90th percentile; ^4^ Sterigenics in Willowbrook, IL; and ^5^ Sterigenics in Smyrna, GA.

**Table 4:** Ambient air EO concentrations (ppb) at the NATTS and UAT locations (as reported by EPA in 2020 and summarized by ATSDR [5]) and at representative background locations associated with the eight EO-emitting industrial facilities.

NATTS / UAT Background		Facility Background	
Location	ppb	Location	ppb ^1^
Phoenix, AZ	0.22	Denver, CO area	0.18
Phoenix, AZ	0.12	Denver, CO area	0.07
Grand Junction, CO	0.17	Denver, CO area	0.25
Pinellas Park, FL	0.08	Denver, CO area	0.08
St. Petersburg, FL	0.08	General Coffee, GA	0.20
Valrico, FL	0.08	South DeKalb, GA	0.19
Northbrook, IL	0.15	South DeKalb, GA	0.15
Schiller Park, IL	0.19	Chicago, IL area	0.18
Ashland, KY	0.16	Chicago, IL area	0.15
Calvert City, KY	0.16	Chicago, IL area	0.09
Grayson, KY	0.14	Chicago, IL area	0.14
Smithland, KY	0.17	Chicago, IL area	0.17
Albany, NY	0.10	Chicago, IL area	0.10
Bronx, NY	0.09	Chicago, IL area	0.11
Bronx, NY	0.08	Chicago, IL area	0.10
Camden, NJ	0.19	Chicago, IL area	0.10
Chester, NJ	0.19	Chicago, IL area	0.10
E. Brunswick, NJ	0.17	Chicago, IL area	0.49
Elizabeth, NJ	0.16	Chicago, IL area	0.15
Pinnacle, NY	0.10	Chicago, IL area	0.27
Queens, NY	0.08	Chicago, IL area	0.11
Rochester, NY	0.10	Gurnee, IL	0.11
Dearborn, MI	0.13	Gurnee, IL	0.09
St. Louis, MO	0.15	Grand Rapids, MI	0.10
Bountiful, UT	0.14	Grand Rapids, MI	0.10
Lacey, WA	0.11	Grand Rapids, MI	0.12
Seattle, WA	0.09	Grand Rapids, MI	0.10
Overall mean ± SD	0.13 ± 0.04	Overall mean ± SD	0.14 ± 0.14

^1^ All values are means of multiple measurements at a single sampling site over time, except for those in Grand Rapids, MI and one location in Chicago, IL, which are single measurements.

## Data Availability

Publicly available datasets were analyzed in this study. These data can be found in the References section.

## Supplementary Materials

The following are available online at https://www.mdpi.com/1660-4601/18/2/607/s1. S1: Publicly available datasets (as of 19 October 2020) concerning ambient EO concentrations in the vicinity of eight sterilization and other EO-emitting industrial facilities and at corresponding representative background locations.
