# Supplementary material for: Ethylene Oxide Exposure in U.S. Populations Residing Near Sterilization and Other Industrial Facilities: Context Based on Endogenous and Total Equivalent Concentration Exposures
Source: Int J Environ Res Public Health. 2021 Jan 12;18(2):607. doi: 10.3390/ijerph18020607 (PMC7828163; doi:10.3390/ijerph18020607)
Supplement: Supplementary file 1 [file ijerph-18-00607-s001.pdf]

Site	Background	Date	Phase	Time	Conc_ppb	LOD_ppb
M1	N	June 6 – 7, 2019	1	Pre shutdown	0.11	
M1	N	June 7 – 8, 2019	1	Pre shutdown	0.1	
M1	N	June 9 – 10, 2019	1	Pre shutdown	0.12	
M1	N	June 12 – 13, 2019	1	Pre shutdown	0.33	
M1	N	June 12 – 13, 2019	1	Pre shutdown	0.21	
M1	N	June 14 – 15, 2019	1	Pre shutdown	2.39	
M1	N	June 15 – 16, 2019	1	Pre shutdown	1.14	
M1	N	June 15 – 16, 2019	1	Pre shutdown	0.077	
M1	N	June 17 – 18, 2019	1	Pre shutdown	ND	0.025
M1	N	June 18 – 19, 2019	1	Pre shutdown	ND	0.025
M1	N	June 21 – 22, 2019	1	Pre shutdown	ND	0.025
M1	N	June 21 – 22, 2019	1	Pre shutdown	ND	0.040
M1	N	June 24 – 25, 2019	1	Pre shutdown	0.484	
M1	N	June 26 – 27, 2019	1	Pre shutdown	0.406	
M1	N	June 27 – 28, 2019	1	Pre shutdown	ND	0.025
M1	N	June 27 – 28, 2019	1	Pre shutdown	0.099	
M1	N	June 27 – 28, 2019	1	Pre shutdown	0.14	
M1	N	June 30 – July 1, 2019	1	Pre shutdown	ND	0.025
M1	N	July 3 – 4, 2019	1	Pre shutdown	0.198	
M2	N	June 6 – 7, 2019	1	Pre shutdown	0.068	
M2	N	June 9 – 10, 2019	1	Pre shutdown	0.081	
M2	N	June 12 – 13, 2019	1	Pre shutdown	0.16	
M2	N	June 12 – 13, 2019	1	Pre shutdown	0.14	
M2	N	June 15 – 16, 2019	1	Pre shutdown	ND	0.025
M2	N	June 18 – 19, 2019	1	Pre shutdown	0.135	
M2	N	June 21 – 22, 2019	1	Pre shutdown	ND	0.025
M2	N	June 21 – 22, 2019	1	Pre shutdown	ND	0.040
M2	N	June 24 – 25, 2019	1	Pre shutdown	ND	0.025
M2	N	June 24 – 25, 2019	1	Pre shutdown	0.16	
M2	N	June 27 – 28, 2019	1	Pre shutdown	ND	0.025
M2	N	June 27 – 28, 2019	1	Pre shutdown	ND	0.040
M2	N	June 30 – July 1, 2019	1	Pre shutdown	ND	0.025
M2	N	July 3 – 4, 2019	1	Pre shutdown	ND	0.025
M3	N	June 6 – 7, 2019	1	Pre shutdown	3.8	
M3	N	June 7 – 8, 2019	1	Pre shutdown	5.6	
M3	N	June 9 – 10, 2019	1	Pre shutdown	1.3	
M3	N	June 12 – 13, 2019	1	Pre shutdown	0.24	
M3	N	June 12 – 13, 2019	1	Pre shutdown	0.26	
M3	N	June 14 – 15, 2019	1	Pre shutdown	ND	0.025
M3	N	June 15 – 16, 2019	1	Pre shutdown	0.686	
M3	N	June 17 – 18, 2019	1	Pre shutdown	2.98	
M3	N	June 17 – 18, 2019	1	Pre shutdown	3.2	
M3	N	June 18 – 19, 2019	1	Pre shutdown	1.75	
M3	N	June 21 – 22, 2019	1	Pre shutdown	3.08	
M3	N	June 21 – 22, 2019	1	Pre shutdown	2.9	
M3	N	June 21 – 22, 2019	1	Pre shutdown	2.8	
M3	N	June 24 – 25, 2019	1	Pre shutdown	0.427	
M3	N	June 26 – 27, 2019	1	Pre shutdown	0.706	
M3	N	June 27 – 28, 2019	1	Pre shutdown	0.683	
M3	N	June 27 – 28, 2019	1	Pre shutdown	0.42	
M3	N	June 30 – July 1, 2019	1	Pre shutdown	0.704	
M3	N	July 3 – 4, 2019	1	Pre shutdown	2.34	
M4	N	June 6 – 7, 2019	1	Pre shutdown	0.081	
M4	N	June 9 – 10, 2019	1	Pre shutdown	0.16	

M4	N	June 12 – 13, 2019	1	Pre shutdown	0.23	
M4	N	June 12 – 13, 2019	1	Pre shutdown	0.23	
M4	N	June 12 – 13, 2019	1	Pre shutdown	0.25	
M4	N	June 18 – 19, 2019	1	Pre shutdown	ND	0.025
M4	N	June 21 – 22, 2019	1	Pre shutdown	ND	0.025
M4	N	June 21 – 22, 2019	1	Pre shutdown	ND	0.040
M4	N	June 21 – 22, 2019	1	Pre shutdown	ND	0.040
M4	N	June 24 – 25, 2019	1	Pre shutdown	ND	0.025
M4	N	June 27 – 28, 2019	1	Pre shutdown	ND	0.025
M4	N	June 27 – 28, 2019	1	Pre shutdown	0.16	
M4	N	June 30 – July 1, 2019	1	Pre shutdown	0.341	
M4	N	July 3 – 4, 2019	1	Pre shutdown	ND	0.025
M5	N	July 3 – 4, 2019	1	Pre shutdown	0.439	
M5	N	July 3 – 4, 2019	1	Pre shutdown	0.25	
R1	Y	June 6 – 7, 2019	1	Pre shutdown	0.068	
R1	Y	June 9 – 10, 2019	1	Pre shutdown	0.07	
R1	Y	June 12 – 13, 2019	1	Pre shutdown	0.053	
R1	Y	June 15 – 16, 2019	1	Pre shutdown	ND	0.025
R1	Y	June 18 – 19, 2019	1	Pre shutdown	ND	0.025
R1	Y	June 21 – 22, 2019	1	Pre shutdown	0.277	
R1	Y	June 24 – 25, 2019	1	Pre shutdown	ND	0.025
R1	Y	June 27 – 28, 2019	1	Pre shutdown	ND	0.025
R1	Y	June 30 – July 1, 2019	1	Pre shutdown	ND	0.025
R1	Y	July 3 – 4, 2019	1	Pre shutdown	0.322	
R2	Y	June 6 – 7, 2019	1	Pre shutdown	0.082	
R2	Y	June 9 – 10, 2019	1	Pre shutdown	0.062	
R2	Y	June 12 – 13, 2019	1	Pre shutdown	0.065	
R2	Y	June 15 – 16, 2019	1	Pre shutdown	0.0896	
R2	Y	June 18 – 19, 2019	1	Pre shutdown	0.708	
R2	Y	June 21 – 22, 2019	1	Pre shutdown	ND	0.025
R2	Y	June 24 – 25, 2019	1	Pre shutdown	ND	0.025
R2	Y	June 27 – 28, 2019	1	Pre shutdown	ND	0.025
R2	Y	June 30 – July 1, 2019	1	Pre shutdown	ND	0.025
R2	Y	July 3 – 4, 2019	1	Pre shutdown	ND	0.025
V1	N	June 6 – 7, 2019	1	Pre shutdown	0.12	
V1	N	June 7 – 8, 2019	1	Pre shutdown	0.062	
V1	N	June 9 – 10, 2019	1	Pre shutdown	0.093	
V1	N	June 9 – 10, 2019	1	Pre shutdown	0.15	
V1	N	June 12 – 13, 2019	1	Pre shutdown	0.11	
V1	N	June 12 – 13, 2019	1	Pre shutdown	0.13	
V1	N	June 14 – 15, 2019	1	Pre shutdown	ND	0.025
V1	N	June 15 – 16, 2019	1	Pre shutdown	ND	0.025
V1	N	June 15 – 16, 2019	1	Pre shutdown	0.082	
V1	N	June 17 – 18, 2019	1	Pre shutdown	ND	0.025
V1	N	June 18 – 19, 2019	1	Pre shutdown	ND	0.025
V1	N	June 18 – 19, 2019	1	Pre shutdown	0.13	
V1	N	June 18 – 19, 2019	1	Pre shutdown	0.1	
V1	N	June 18 – 19, 2019	1	Pre shutdown	0.089	
V1	N	June 21 – 22, 2019	1	Pre shutdown	0.495	
V1	N	June 21 – 22, 2019	1	Pre shutdown	ND	0.040
V1	N	June 24 – 25, 2019	1	Pre shutdown	ND	0.025
V1	N	June 24 – 25, 2019	1	Pre shutdown	ND	0.025
V1	N	June 24 – 25, 2019	1	Pre shutdown	0.075	
V1	N	June 26 – 27, 2019	1	Pre shutdown	0.102	
V1	N	June 27 – 28, 2019	1	Pre shutdown	0.126	

V1	N	June 27 – 28, 2019	1	Pre shutdown	ND	0.0173
V1	N	June 30 – July 1, 2019	1	Pre shutdown	0.0853	
V1	N	June 30 – July 1, 2019	1	Pre shutdown	0.0608	
V1	N	June 30 – July 1, 2019	1	Pre shutdown	0.14	
V1	N	July 3 – 4, 2019	1	Pre shutdown	ND	0.025
V1	N	July 3 – 4, 2019	1	Pre shutdown	0.0674	
V1	N	July 6 – 7, 2019	1	Pre shutdown	0.0579	
V2	N	June 6 – 7, 2019	1	Pre shutdown	0.076	
V2	N	June 9 – 10, 2019	1	Pre shutdown	0.079	
V2	N	June 9 – 10, 2019	1	Pre shutdown	0.14	
V2	N	June 12 – 13, 2019	1	Pre shutdown	0.11	
V2	N	June 12 – 13, 2019	1	Pre shutdown	0.23	
V2	N	June 12 – 13, 2019	1	Pre shutdown	0.12	
V2	N	June 15 – 16, 2019	1	Pre shutdown	ND	0.025
V2	N	June 15 – 16, 2019	1	Pre shutdown	0.14	
V2	N	June 18 – 19, 2019	1	Pre shutdown	ND	0.025
V2	N	June 18 – 19, 2019	1	Pre shutdown	0.19	
V2	N	June 21 – 22, 2019	1	Pre shutdown	ND	0.025
V2	N	June 21 – 22, 2019	1	Pre shutdown	0.049	
V2	N	June 24 – 25, 2019	1	Pre shutdown	ND	0.025
V2	N	June 24 – 25, 2019	1	Pre shutdown	0.22	
V2	N	June 27 – 28, 2019	1	Pre shutdown	ND	0.025
V2	N	June 27 – 28, 2019	1	Pre shutdown	0.145	
V2	N	June 27 – 28, 2019	1	Pre shutdown	0.255	
V2	N	June 30 – July 1, 2019	1	Pre shutdown	0.321	
V2	N	June 30 – July 1, 2019		Pre shutdown	0.206	
V2	N	July 3 – 4, 2019	1	Pre shutdown	0.155	
V2	N	July 3 – 4, 2019		Pre shutdown	0.0392	
V2	N	July 6 – 7, 2019	1	Pre shutdown	0.109	
V2	N	July 6 – 7, 2019		Pre shutdown	0.112	
V3	N	June 6 – 7, 2019	1	Pre shutdown	0.15	
V3	N	June 7 – 8, 2019	1	Pre shutdown	0.34	
V3	N	June 9 – 10, 2019	1	Pre shutdown	0.068	
V3	N	June 12 – 13, 2019	1	Pre shutdown	0.13	
V3	N	June 14 – 15, 2019	1	Pre shutdown	ND	0.025
V3	N	June 15 – 16, 2019	1	Pre shutdown	0.608	
V3	N	June 17 – 18, 2019	1	Pre shutdown	ND	0.025
V3	N	June 18 – 19, 2019	1	Pre shutdown	ND	0.025
V3	N	June 21 – 22, 2019	1	Pre shutdown	ND	0.025
V3	N	June 24 – 25, 2019	1	Pre shutdown	0.153	
V3	N	June 26 – 27, 2019	1	Pre shutdown	0.147	
V3	N	June 26 – 27, 2019	1	Pre shutdown	0.11	
V3	N	June 27 – 28, 2019	1	Pre shutdown	ND	0.025
V3	N	June 30 – July 1, 2019	1	Pre shutdown	0.209	
V3	N	July 3 – 4, 2019	1	Pre shutdown	0.192	
V4	N	June 6 – 7, 2019	1	Pre shutdown	0.11	
V4	N	June 9 – 10, 2019	1	Pre shutdown	0.07	
V4	N	June 12 – 13, 2019	1	Pre shutdown	0.18	
V4	N	June 15 – 16, 2019	1	Pre shutdown	ND	0.025
V4	N	June 18 – 19, 2019	1	Pre shutdown	ND	0.025
V4	N	June 21 – 22, 2019	1	Pre shutdown	ND	0.025
V4	N	June 24 – 25, 2019	1	Pre shutdown	ND	0.025
V4	N	June 30 – July 1, 2019	1	Pre shutdown	0.119	
V4	N	June 30 – July 1, 2019	1	Pre shutdown	2	
V4	N	July 3 – 4, 2019	1	Pre shutdown	ND	0.025

M1	N	November 1 - 2, 2019	2	Pre shutdown	0.185	
M1	N	November 4 - 5, 2019	2	Pre shutdown	0.0872	
M1	N	November 7 - 8, 2019	2	Pre shutdown	ND	0.025
M1	N	November 10 - 11, 2019	2	Pre shutdown	0.044	
M1	N	November 13 - 14, 2019	2	Pre shutdown	0.218	
M1	N	November 16 - 17, 2019	2	Pre shutdown	0.0636	
M1	N	November 19 - 20, 2019	2	Pre shutdown	0.0723	
M1	N	November 22 - 23, 2019	2	Pre shutdown	0.161	
M1	N	November 25 - 26, 2019	2	Pre shutdown	0.175	
M1	N	November 28 - 29, 2019	2	Pre shutdown	0.0444	
M1	N	December 1 - 2, 2019	2	Pre shutdown	0.0468	
M1	N	December 4 - 5, 2019	2	Pre shutdown	0.569	
M1	N	December 7 - 8, 2019	2	Pre shutdown	0.0689	
M1	N	December 10 - 11, 2019	2	Pre shutdown	0.0352	
M1	N	December 13 - 14, 2019	2	Shutdown	0.074	
M1	N	December 16 - 17, 2019	2	Shutdown	0.0343	
M1	N	December 19 - 20, 2019	2	Shutdown	0.133	
M1	N	December 22 - 23, 2019	2	Shutdown	0.107	
M1	N	December 25 - 26, 2019	2	Shutdown	0.0432	
M1	N	December 28 - 29, 2019	2	Shutdown	0.112	
M1	N	Dec 31, 2019 - Jan 1, 2020	2	Shutdown	0.0755	
M1	N	January 3 - 4, 2020	2	Shutdown	0.161	
M1	N	January 6 - 7, 2020	2	Shutdown	0.0917	
M1	N	January 9 - 10, 2020	2	Shutdown	0.0495	
M1	N	January 12 - 13, 2020	2	Shutdown	0.0164	
M1	N	January 15 - 16, 2020	2	Shutdown	0.0376	
M1	N	January 18 - 19, 2020	2	Shutdown	0.104	
M1	N	January 21 - 22, 2020	2	Shutdown	0.131	
M2	N	October 26 - 27, 2019	2	Pre shutdown	0.0359	
M2	N	October 29 - 30, 2019	2	Pre shutdown	0.0313	
M2	N	November 1 - 2, 2019	2	Pre shutdown	0.161	
M2	N	November 4 - 5, 2019	2	Pre shutdown	ND	0.025
M2	N	November 7 - 8, 2019	2	Pre shutdown	0.0847	
M2	N	November 10 - 11, 2019	2	Pre shutdown	ND	0.025
M2	N	November 13 - 14, 2019	2	Pre shutdown	0.0631	
M2	N	November 16 - 17, 2019	2	Pre shutdown	0.0671	
M2	N	November 19 - 20, 2019	2	Pre shutdown	0.109	
M2	N	November 22 - 23, 2019	2	Pre shutdown	0.344	
M2	N	November 25 - 26, 2019	2	Pre shutdown	0.132	
M2	N	November 28 - 29, 2019	2	Pre shutdown	0.0519	
M2	N	December 1 - 2, 2019	2	Pre shutdown	0.0514	
M2	N	December 4 - 5, 2019	2	Pre shutdown	0.117	
M2	N	December 7 - 8, 2019	2	Pre shutdown	0.0757	
M2	N	December 10 - 11, 2019	2	Pre shutdown	0.516	
M2	N	December 13 - 14, 2019	2	Shutdown	0.0377	
M2	N	December 16 - 17, 2019	2	Shutdown	0.0803	
M2	N	December 19 - 20, 2019	2	Shutdown	0.0776	
M2	N	December 22 - 23, 2019	2	Shutdown	0.0593	
M2	N	December 25 - 26, 2019	2	Shutdown	0.0863	
M2	N	December 28 - 29, 2019	2	Shutdown	0.0385	
M2	N	Dec 31, 2019 - Jan 1, 2020	2	Shutdown	0.048	
M2	N	January 3 - 4, 2020	2	Shutdown	0.0338	
M2	N	January 6 - 7, 2020	2	Shutdown	0.0563	
M2	N	January 9 - 10, 2020	2	Shutdown	0.0439	
M2	N	January 12 - 13, 2020	2	Shutdown	0.0737	

M2	N	January 15 - 16, 2020	2	Shutdown	0.0606
M2	N	January 18 - 19, 2020	2	Shutdown	0.0596
M2	N	January 21 - 22, 2020	2	Shutdown	0.127
M3	N	October 26 - 27, 2019	2	Pre shutdown	0.89
M3	N	October 29 - 30, 2019	2	Pre shutdown	0.121
M3	N	November 1 - 2, 2019	2	Pre shutdown	0.0597
M3	N	November 4 - 5, 2019	2	Pre shutdown	0.0525
M3	N	November 7 - 8, 2019	2	Pre shutdown	0.146
M3	N	November 10 - 11, 2019	2	Pre shutdown	0.0378
M3	N	November 13 - 14, 2019	2	Pre shutdown	0.0328
M3	N	November 16 - 17, 2019	2	Pre shutdown	0.0645
M3	N	November 19 - 20, 2019	2	Pre shutdown	0.0412
M3	N	November 22 - 23, 2019	2	Pre shutdown	0.0882
M3	N	November 25 - 26, 2019	2	Pre shutdown	0.11
M3	N	November 28 - 29, 2019	2	Pre shutdown	0.038
M3	N	December 1 - 2, 2019	2	Pre shutdown	0.107
M3	N	December 4 - 5, 2019	2	Pre shutdown	0.0532
M3	N	December 7 - 8, 2019	2	Pre shutdown	0.0503
M3	N	December 10 - 11, 2019	2	Pre shutdown	0.067
M3	N	December 13 - 14, 2019	2	Shutdown	0.116
M3	N	December 16 - 17, 2019	2	Shutdown	0.0661
M3	N	December 19 - 20, 2019	2	Shutdown	0.148
M3	N	December 22 - 23, 2019	2	Shutdown	0.0707
M3	N	December 25 - 26, 2019	2	Shutdown	0.0482
M3	N	December 28 - 29, 2019	2	Shutdown	0.0344
M3	N	Dec 31, 2019 - Jan 1, 2020	2	Shutdown	0.132
M3	N	January 3 - 4, 2020	2	Shutdown	0.0627
M3	N	January 6 - 7, 2020	2	Shutdown	0.0586
M3	N	January 9 - 10, 2020	2	Shutdown	0.0379
M3	N	January 12 - 13, 2020	2	Shutdown	0.0723
M3	N	January 15 - 16, 2020	2	Shutdown	0.0542
M3	N	January 18 - 19, 2020	2	Shutdown	0.0993
M3	N	January 21 - 22, 2020	2	Shutdown	0.144
M4	N	October 26 - 27, 2019	2	Pre shutdown	0.21
M4	N	October 29 - 30, 2019	2	Pre shutdown	0.0626
M4	N	November 1 - 2, 2019	2	Pre shutdown	0.31
M4	N	November 4 - 5, 2019	2	Pre shutdown	0.388
M4	N	November 7 - 8, 2019	2	Pre shutdown	0.0929
M4	N	November 10 - 11, 2019	2	Pre shutdown	0.0538
M4	N	November 13 - 14, 2019	2	Pre shutdown	0.0812
M4	N	November 16 - 17, 2019	2	Pre shutdown	0.0797
M4	N	November 19 - 20, 2019	2	Pre shutdown	0.296
M4	N	November 22 - 23, 2019	2	Pre shutdown	0.178
M4	N	November 25 - 26, 2019	2	Pre shutdown	0.335
M4	N	November 28 - 29, 2019	2	Pre shutdown	0.0444
M4	N	December 1 - 2, 2019	2	Pre shutdown	0.134
M4	N	December 4 - 5, 2019	2	Pre shutdown	0.289
M4	N	December 7 - 8, 2019	2	Pre shutdown	0.0424
M4	N	December 10 - 11, 2019	2	Pre shutdown	0.414
M4	N	December 13 - 14, 2019	2	Shutdown	0.0962
M4	N	December 16 - 17, 2019	2	Shutdown	0.0263
M4	N	December 19 - 20, 2019	2	Shutdown	0.131
M4	N	December 22 - 23, 2019	2	Shutdown	0.0964
M4	N	December 25 - 26, 2019	2	Shutdown	0.112
M4	N	January 3 - 4, 2020	2	Shutdown	0.104

M4	N	January 6 - 7, 2020	2	Shutdown	0.0539	
M4	N	January 9 - 10, 2020	2	Shutdown	0.0912	
M4	N	January 12 - 13, 2020	2	Shutdown	0.0608	
M4	N	January 15 - 16, 2020	2	Shutdown	0.101	
M4	N	January 18 - 19, 2020	2	Shutdown	0.128	
M4	N	January 21 - 22, 2020	2	Shutdown	0.0597	
M5	N	October 26 - 27, 2019	2	Pre shutdown	0.0856	
M5	N	October 29 - 30, 2019	2	Pre shutdown	0.118	
M5	N	November 1 - 2, 2019	2	Pre shutdown	0.0361	
M5	N	November 4 - 5, 2019	2	Pre shutdown	ND	0.025
M5	N	November 7 - 8, 2019	2	Pre shutdown	ND	0.025
M5	N	November 10 - 11, 2019	2	Pre shutdown	0.0359	
M5	N	November 13 - 14, 2019	2	Pre shutdown	0.0264	
M5	N	November 16 - 17, 2019	2	Pre shutdown	0.446	
M5	N	November 19 - 20, 2019	2	Pre shutdown	0.0549	
M5	N	November 22 - 23, 2019	2	Pre shutdown	0.0906	
M5	N	November 25 - 26, 2019	2	Pre shutdown	0.147	
M5	N	November 28 - 29, 2019	2	Pre shutdown	0.114	
M5	N	December 1 - 2, 2019	2	Pre shutdown	0.11	
M5	N	December 4 - 5, 2019	2	Pre shutdown	0.0782	
M5	N	December 7 - 8, 2019	2	Pre shutdown	0.081	
M5	N	December 10 - 11, 2019	2	Pre shutdown	0.0187	
M5	N	December 13 - 14, 2019	2	Shutdown	0.0786	
M5	N	December 16 - 17, 2019	2	Shutdown	0.131	
M5	N	December 19 - 20, 2019	2	Shutdown	0.0716	
M5	N	December 22 - 23, 2019	2	Shutdown	0.112	
M5	N	December 25 - 26, 2019	2	Shutdown	0.132	
M5	N	December 28 - 29, 2019	2	Shutdown	0.169	
M5	N	Dec 31, 2019 - Jan 1, 2020	2	Shutdown	0.101	
M5	N	January 3 - 4, 2020	2	Shutdown	0.163	
M5	N	January 6 - 7, 2020	2	Shutdown	0.0266	
M5	N	January 9 - 10, 2020	2	Shutdown	0.134	
M5	N	January 12 - 13, 2020	2	Shutdown	0.0915	
M5	N	January 15 - 16, 2020	2	Shutdown	0.127	
M5	N	January 18 - 19, 2020	2	Shutdown	0.114	
M5	N	January 21 - 22, 2020	2	Shutdown	0.129	
R1	Y	October 26 - 27, 2019	2	Pre shutdown	0.0994	
R1	Y	October 29 - 30, 2019	2	Pre shutdown	0.0968	
R1	Y	November 1 - 2, 2019	2	Pre shutdown	0.114	
R1	Y	November 4 - 5, 2019	2	Pre shutdown	0.0859	
R1	Y	November 7 - 8, 2019	2	Pre shutdown	0.0655	
R1	Y	November 10 - 11, 2019	2	Pre shutdown	0.0377	
R1	Y	November 13 - 14, 2019	2	Pre shutdown	0.0991	
R1	Y	November 16 - 17, 2019	2	Pre shutdown	0.178	
R1	Y	November 19 - 20, 2019	2	Pre shutdown	0.112	
R1	Y	November 22 - 23, 2019	2	Pre shutdown	0.0966	
R1	Y	November 25 - 26, 2019	2	Pre shutdown	0.095	
R1	Y	November 28 - 29, 2019	2	Pre shutdown	0.134	
R1	Y	December 1 - 2, 2019	2	Pre shutdown	0.0733	
R1	Y	December 4 - 5, 2019	2	Pre shutdown	0.115	
R1	Y	December 7 - 8, 2019	2	Pre shutdown	0.0706	
R1	Y	December 10 - 11, 2019	2	Pre shutdown	0.0366	
R1	Y	December 13 - 14, 2019	2	Shutdown	0.0487	
R1	Y	December 16 - 17, 2019	2	Shutdown	0.084	
R1	Y	December 19 - 20, 2019	2	Shutdown	0.114	

R1	Y	December 22 - 23, 2019	2	Shutdown	0.0881	
R1	Y	December 25 - 26, 2019	2	Shutdown	0.128	
R1	Y	December 28 - 29, 2019	2	Shutdown	0.281	
R1	Y	Dec 31, 2019 - Jan 1, 2020	2	Shutdown	0.0613	
R1	Y	January 3 - 4, 2020	2	Shutdown	0.0747	
R1	Y	January 6 - 7, 2020	2	Shutdown	0.125	
R1	Y	January 9 - 10, 2020	2	Shutdown	0.0611	
R1	Y	January 12 - 13, 2020	2	Shutdown	0.0244	
R1	Y	January 15 - 16, 2020	2	Shutdown	0.106	
R1	Y	January 18 - 19, 2020	2	Shutdown	0.0667	
R1	Y	January 21 - 22, 2020	2	Shutdown	0.175	
R2	Y	October 26 - 27, 2019	2	Pre shutdown	0.104	
R2	Y	October 29 - 30, 2019	2	Pre shutdown	0.0344	
R2	Y	November 1 - 2, 2019	2	Pre shutdown	ND	0.025
R2	Y	November 4 - 5, 2019	2	Pre shutdown	0.0628	
R2	Y	November 7 - 8, 2019	2	Pre shutdown	0.0188	
R2	Y	November 10 - 11, 2019	2	Pre shutdown	0.0689	
R2	Y	November 13 - 14, 2019	2	Pre shutdown	0.0373	
R2	Y	November 16 - 17, 2019	2	Pre shutdown	0.0777	
R2	Y	November 19 - 20, 2019	2	Pre shutdown	0.155	
R2	Y	November 22 - 23, 2019	2	Pre shutdown	0.0968	
R2	Y	November 25 - 26, 2019	2	Pre shutdown	0.134	
R2	Y	November 28 - 29, 2019	2	Pre shutdown	0.135	
R2	Y	December 1 - 2, 2019	2	Pre shutdown	0.067	
R2	Y	December 4 - 5, 2019	2	Pre shutdown	0.0357	
R2	Y	December 7 - 8, 2019	2	Pre shutdown	0.0414	
R2	Y	December 10 - 11, 2019	2	Pre shutdown	0.0246	
R2	Y	December 13 - 14, 2019	2	Shutdown	0.0646	
R2	Y	December 16 - 17, 2019	2	Shutdown	0.0924	
R2	Y	December 19 - 20, 2019	2	Shutdown	0.036	
R2	Y	December 22 - 23, 2019	2	Shutdown	0.0335	
R2	Y	December 25 - 26, 2019	2	Shutdown	0.0981	
R2	Y	December 28 - 29, 2019	2	Shutdown	0.141	
R2	Y	Dec 31, 2019 - Jan 1, 2020	2	Shutdown	0.0322	
R2	Y	January 3 - 4, 2020	2	Shutdown	0.0376	
R2	Y	January 6 - 7, 2020	2	Shutdown	0.166	
R2	Y	January 9 - 10, 2020	2	Shutdown	0.0266	
R2	Y	January 12 - 13, 2020	2	Shutdown	0.0467	
R2	Y	January 15 - 16, 2020	2	Shutdown	0.0429	
R2	Y	January 18 - 19, 2020	2	Shutdown	0.0589	
R2	Y	January 21 - 22, 2020	2	Shutdown	0.0486	
V1	N	October 26 - 27, 2019	2	Pre shutdown	0.146	
V1	N	October 29 - 30, 2019	2	Pre shutdown	0.167	
V1	N	November 1 - 2, 2019	2	Pre shutdown	0.223	
V1	N	November 4 - 5, 2019	2	Pre shutdown	0.0601	
V1	N	November 7 - 8, 2019	2	Pre shutdown	0.0462	
V1	N	November 10 - 11, 2019	2	Pre shutdown	0.03	
V1	N	November 13 - 14, 2019	2	Pre shutdown	2.93	
V1	N	November 16 - 17, 2019	2	Pre shutdown	0.125	
V1	N	November 19 - 20, 2019	2	Pre shutdown	0.118	
V1	N	November 22 - 23, 2019	2	Pre shutdown	0.219	
V1	N	November 25 - 26, 2019	2	Pre shutdown	0.0832	
V1	N	November 28 - 29, 2019	2	Pre shutdown	0.0454	
V1	N	December 1 - 2, 2019	2	Pre shutdown	0.0443	
V1	N	December 4 - 5, 2019	2	Pre shutdown	0.128	

V1	N	December 7 - 8, 2019	2	Pre shutdown	0.0582	
V1	N	December 10 - 11, 2019	2	Pre shutdown	0.0307	
V1	N	December 13 - 14, 2019	2	Shutdown	0.0499	
V1	N	December 16 - 17, 2019	2	Shutdown	0.0314	
V1	N	December 19 - 20, 2019	2	Shutdown	0.111	
V1	N	December 22 - 23, 2019	2	Shutdown	0.128	
V1	N	December 25 - 26, 2019	2	Shutdown	0.109	
V1	N	December 28 - 29, 2019	2	Shutdown	0.0714	
V1	N	January 3 - 4, 2020	2	Shutdown	0.143	
V1	N	January 6 - 7, 2020	2	Shutdown	0.0606	
V1	N	January 9 - 10, 2020	2	Shutdown	0.0641	
V1	N	January 12 - 13, 2020	2	Shutdown	0.0357	
V1	N	January 15 - 16, 2020	2	Shutdown	0.0379	
V1	N	January 18 - 19, 2020	2	Shutdown	0.0625	
V1	N	January 21 - 22, 2020	2	Shutdown	ND	0.025
V2	N	October 29 - 30, 2019	2	Pre shutdown	0.138	
V2	N	November 1 - 2, 2019	2	Pre shutdown	2.03	
V2	N	November 4 - 5, 2019	2	Pre shutdown	0.743	
V2	N	November 7 - 8, 2019	2	Pre shutdown	0.0836	
V2	N	November 10 - 11, 2019	2	Pre shutdown	0.016	
V2	N	November 13 - 14, 2019	2	Pre shutdown	4.91	
V2	N	November 16 - 17, 2019	2	Pre shutdown	0.0293	
V2	N	November 22 - 23, 2019	2	Pre shutdown	0.125	
V2	N	November 25 - 26, 2019	2	Pre shutdown	0.197	
V2	N	November 28 - 29, 2019	2	Pre shutdown	0.0471	
V2	N	December 1 - 2, 2019	2	Pre shutdown	0.0765	
V2	N	December 4 - 5, 2019	2	Pre shutdown	0.464	
V2	N	December 7 - 8, 2019	2	Pre shutdown	0.0898	
V2	N	December 10 - 11, 2019	2	Pre shutdown	0.311	
V2	N	December 13 - 14, 2019	2	Shutdown	0.0416	
V2	N	December 16 - 17, 2019	2	Shutdown	0.105	
V2	N	December 19 - 20, 2019	2	Shutdown	0.0743	
V2	N	December 22 - 23, 2019	2	Shutdown	0.0353	
V2	N	December 25 - 26, 2019	2	Shutdown	0.0947	
V2	N	December 28 - 29, 2019	2	Shutdown	0.0471	
V2	N	Dec 31, 2019 - Jan 1, 2020	2	Shutdown	0.196	
V2	N	January 3 - 4, 2020	2	Shutdown	0.142	
V2	N	January 6 - 7, 2020	2	Shutdown	0.291	
V2	N	January 9 - 10, 2020	2	Shutdown	0.0241	
V2	N	January 12 - 13, 2020	2	Shutdown	0.0982	
V2	N	January 15 - 16, 2020	2	Shutdown	0.0629	
V2	N	January 18 - 19, 2020	2	Shutdown	0.103	
V2	N	January 21 - 22, 2020	2	Shutdown	0.108	
V3	N	October 26 - 27, 2019	2	Pre shutdown	0.0661	
V3	N	October 29 - 30, 2019	2	Pre shutdown	0.184	
V3	N	November 1 - 2, 2019	2	Pre shutdown	0.0667	
V3	N	November 4 - 5, 2019	2	Pre shutdown	0.123	
V3	N	November 7 - 8, 2019	2	Pre shutdown	0.0277	
V3	N	November 10 - 11, 2019	2	Pre shutdown	1.12	
V3	N	November 13 - 14, 2019	2	Pre shutdown	0.0254	
V3	N	November 16 - 17, 2019	2	Pre shutdown	0.0879	
V3	N	November 19 - 20, 2019	2	Pre shutdown	0.106	
V3	N	November 22 - 23, 2019	2	Pre shutdown	0.0272	
V3	N	November 25 - 26, 2019	2	Pre shutdown	0.158	
V3	N	November 28 - 29, 2019	2	Pre shutdown	0.707	

V3	N	December 1 - 2, 2019	2	Pre shutdown	0.125	
V3	N	December 4 - 5, 2019	2	Pre shutdown	0.0975	
V3	N	December 7 - 8, 2019	2	Pre shutdown	0.0937	
V3	N	December 10 - 11, 2019	2	Pre shutdown	0.0846	
V3	N	December 16 - 17, 2019	2	Shutdown	0.117	
V3	N	December 19 - 20, 2019	2	Shutdown	0.06	
V3	N	December 22 - 23, 2019	2	Shutdown	0.046	
V3	N	December 25 - 26, 2019	2	Shutdown	0.123	
V3	N	December 28 - 29, 2019	2	Shutdown	0.147	
V3	N	Dec 31, 2019 - Jan 1, 2020	2	Shutdown	0.12	
V3	N	January 3 - 4, 2020	2	Shutdown	0.156	
V3	N	January 6 - 7, 2020	2	Shutdown	0.0835	
V3	N	January 9 - 10, 2020	2	Shutdown	0.164	
V3	N	January 12 - 13, 2020	2	Shutdown	0.0351	
V3	N	January 15 - 16, 2020	2	Shutdown	0.0266	
V3	N	January 18 - 19, 2020	2	Shutdown	0.0311	
V3	N	January 21 - 22, 2020	2	Shutdown	0.0849	
V4	N	October 26 - 27, 2019	2	Pre shutdown	ND	0.025
V4	N	October 29 - 30, 2019	2	Pre shutdown	0.092	
V4	N	November 1 - 2, 2019	2	Pre shutdown	0.12	
V4	N	November 4 - 5, 2019	2	Pre shutdown	0.0308	
V4	N	November 7 - 8, 2019	2	Pre shutdown	0.0678	
V4	N	November 10 - 11, 2019	2	Pre shutdown	0.0802	
V4	N	November 13 - 14, 2019	2	Pre shutdown	0.0269	
V4	N	November 16 - 17, 2019	2	Pre shutdown	0.0767	
V4	N	November 19 - 20, 2019	2	Pre shutdown	0.0885	
V4	N	November 22 - 23, 2019	2	Pre shutdown	0.199	
V4	N	November 25 - 26, 2019	2	Pre shutdown	0.0702	
V4	N	November 28 - 29, 2019	2	Pre shutdown	0.159	
V4	N	December 1 - 2, 2019	2	Pre shutdown	0.0741	
V4	N	December 4 - 5, 2019	2	Pre shutdown	0.0468	
V4	N	December 7 - 8, 2019	2	Pre shutdown	0.0882	
V4	N	December 10 - 11, 2019	2	Pre shutdown	0.0512	
V4	N	December 13 - 14, 2019	2	Shutdown	0.057	
V4	N	December 16 - 17, 2019	2	Shutdown	0.0345	
V4	N	December 19 - 20, 2019	2	Shutdown	0.0827	
V4	N	December 22 - 23, 2019	2	Shutdown	0.0441	
V4	N	December 25 - 26, 2019	2	Shutdown	0.032	
V4	N	December 28 - 29, 2019	2	Shutdown	0.0314	
V4	N	Dec 31, 2019 - Jan 1, 2020	2	Shutdown	0.218	
V4	N	January 3 - 4, 2020	2	Shutdown	0.129	
V4	N	January 6 - 7, 2020	2	Shutdown	0.103	
V4	N	January 9 - 10, 2020	2	Shutdown	0.128	
V4	N	January 12 - 13, 2020	2	Shutdown	0.0196	
V4	N	January 15 - 16, 2020	2	Shutdown	0.0278	
V4	N	January 18 - 19, 2020	2	Shutdown	0.123	
V4	N	January 21 - 22, 2020	2	Shutdown	0.0503	
V5	N	October 29 - 30, 2019	2	Pre shutdown	0.0754	
V5	N	November 1 - 2, 2019	2	Pre shutdown	0.122	
V5	N	November 4 - 5, 2019	2	Pre shutdown	0.111	
V5	N	November 7 - 8, 2019	2	Pre shutdown	0.104	
V5	N	November 10 - 11, 2019	2	Pre shutdown	0.0474	
V5	N	November 13 - 14, 2019	2	Pre shutdown	0.0413	
V5	N	November 16 - 17, 2019	2	Pre shutdown	0.15	
V5	N	November 19 - 20, 2019	2	Pre shutdown	0.0587	

V5	N	November 22 - 23, 2019	2	Pre shutdown	0.11
V5	N	November 25 - 26, 2019	2	Pre shutdown	0.0746
V5	N	November 28 - 29, 2019	2	Pre shutdown	0.132
V5	N	December 1 - 2, 2019	2	Pre shutdown	0.0693
V5	N	December 4 - 5, 2019	2	Pre shutdown	0.0678
V5	N	December 7 - 8, 2019	2	Pre shutdown	0.0383
V5	N	December 10 - 11, 2019	2	Pre shutdown	0.0707
V5	N	December 13 - 14, 2019	2	Shutdown	0.0436
V5	N	December 16 - 17, 2019	2	Shutdown	0.0691
V5	N	December 19 - 20, 2019	2	Shutdown	0.48
V5	N	December 22 - 23, 2019	2	Shutdown	0.0227
V5	N	December 25 - 26, 2019	2	Shutdown	0.233
V5	N	December 28 - 29, 2019	2	Shutdown	0.404
V5	N	Dec 31, 2019 - Jan 1, 2020	2	Shutdown	0.112
V5	N	January 3 - 4, 2020	2	Shutdown	0.0716
V5	N	January 6 - 7, 2020	2	Shutdown	0.144
V5	N	January 9 - 10, 2020	2	Shutdown	0.0364
V5	N	January 12 - 13, 2020	2	Shutdown	0.0463
V5	N	January 15 - 16, 2020	2	Shutdown	0.027
V5	N	January 18 - 19, 2020	2	Shutdown	0.145
V5	N	January 21 - 22, 2020	2	Shutdown	0.0344
M1	N	April 7 - 8, 2020	3	Post shutdown (new controls)	0.168
M1	N	April 10 - 11, 2020	3	Post shutdown (new controls)	0.0825
M1	N	April 13 - 14 2020	3	Post shutdown (new controls)	0.277
M1	N	April 16 - 17, 2020	3	Post shutdown (new controls)	0.0952
M1	N	April 19 - 20, 2020	3	Post shutdown (new controls)	0.0706
M1	N	April 22 - 23, 2020	3	Post shutdown (new controls)	0.406
M1	N	April 25 - 26, 2020	3	Post shutdown (new controls)	0.112
M1	N	May 1 - 2, 2020	3	Post shutdown (new controls)	0.508
M2	N	April 7 - 8, 2020	3	Post shutdown (new controls)	0.0549
M2	N	April 13 - 14 2020	3	Post shutdown (new controls)	0.18
M2	N	April 16 - 17, 2020	3	Post shutdown (new controls)	0.218
M2	N	April 19 - 20, 2020	3	Post shutdown (new controls)	0.0645
M2	N	April 22 - 23, 2020	3	Post shutdown (new controls)	0.0395
M2	N	April 25 - 26, 2020	3	Post shutdown (new controls)	0.095
M2	N	May 1 - 2, 2020	3	Post shutdown (new controls)	0.042
M3	N	April 4 - 5, 2020	3	Post shutdown (new controls)	0.142
M3	N	April 7 - 8, 2020	3	Post shutdown (new controls)	0.194
M3	N	April 10 - 11, 2020	3	Post shutdown (new controls)	0.0475
M3	N	April 16 - 17, 2020	3	Post shutdown (new controls)	0.372
M3	N	April 19 - 20, 2020	3	Post shutdown (new controls)	0.0666
M3	N	April 22 - 23, 2020	3	Post shutdown (new controls)	0.311
M3	N	April 28 - 29, 2020	3	Post shutdown (new controls)	0.31
M3	N	May 1 - 2, 2020	3	Post shutdown (new controls)	0.241
M4	N	April 4 - 5, 2020	3	Post shutdown (new controls)	0.0285
M4	N	April 7 - 8, 2020	3	Post shutdown (new controls)	0.106
M4	N	April 10 - 11, 2020	3	Post shutdown (new controls)	0.0834
M4	N	April 13 - 14 2020	3	Post shutdown (new controls)	0.109
M4	N	April 16 - 17, 2020	3	Post shutdown (new controls)	0.423
M4	N	April 19 - 20, 2020	3	Post shutdown (new controls)	0.107
M4	N	April 22 - 23, 2020	3	Post shutdown (new controls)	0.156
M4	N	April 25 - 26, 2020	3	Post shutdown (new controls)	0.0939
M4	N	April 28 - 29, 2020	3	Post shutdown (new controls)	0.265
M4	N	May 1 - 2, 2020	3	Post shutdown (new controls)	0.101
M5	N	April 4 - 5, 2020	3	Post shutdown (new controls)	0.0883

M5	N	April 7 - 8, 2020	3	Post shutdown (new controls)	0.0528
M5	N	April 10 - 11, 2020	3	Post shutdown (new controls)	0.229
M5	N	April 13 - 14 2020	3	Post shutdown (new controls)	0.272
M5	N	April 16 - 17, 2020	3	Post shutdown (new controls)	0.0645
M5	N	April 19 - 20, 2020	3	Post shutdown (new controls)	0.0569
M5	N	April 22 - 23, 2020	3	Post shutdown (new controls)	0.25
M5	N	April 25 - 26, 2020	3	Post shutdown (new controls)	0.201
R1	Y	April 4 - 5, 2020	3	Post shutdown (new controls)	0.0354
R1	Y	April 7 - 8, 2020	3	Post shutdown (new controls)	0.129
R1	Y	April 10 - 11, 2020	3	Post shutdown (new controls)	0.2
R1	Y	April 13 - 14 2020	3	Post shutdown (new controls)	0.0298
R1	Y	April 16 - 17, 2020	3	Post shutdown (new controls)	0.121
R1	Y	April 19 - 20, 2020	3	Post shutdown (new controls)	0.131
R1	Y	April 22 - 23, 2020	3	Post shutdown (new controls)	0.204
R1	Y	April 25 - 26, 2020	3	Post shutdown (new controls)	0.459
R1	Y	May 1 - 2, 2020	3	Post shutdown (new controls)	0.426
R2	Y	April 4 - 5, 2020	3	Post shutdown (new controls)	0.03998
R2	Y	April 7 - 8, 2020	3	Post shutdown (new controls)	0.136
R2	Y	April 10 - 11, 2020	3	Post shutdown (new controls)	0.0437
R2	Y	April 13 - 14 2020	3	Post shutdown (new controls)	0.207
R2	Y	April 16 - 17, 2020	3	Post shutdown (new controls)	0.143
R2	Y	April 19 - 20, 2020	3	Post shutdown (new controls)	0.113
R2	Y	April 22 - 23, 2020	3	Post shutdown (new controls)	0.0541
R2	Y	April 25 - 26, 2020	3	Post shutdown (new controls)	0.0482
R2	Y	May 1 - 2, 2020	3	Post shutdown (new controls)	0.459
V1	N	April 4 - 5, 2020	3	Post shutdown (new controls)	0.0313
V1	N	April 7 - 8, 2020	3	Post shutdown (new controls)	0.0916
V1	N	April 10 - 11, 2020	3	Post shutdown (new controls)	0.292
V1	N	April 13 - 14 2020	3	Post shutdown (new controls)	0.2
V1	N	April 16 - 17, 2020	3	Post shutdown (new controls)	0.392
V1	N	April 22 - 23, 2020	3	Post shutdown (new controls)	0.259
V1	N	April 25 - 26, 2020	3	Post shutdown (new controls)	0.296
V1	N	April 28 - 29, 2020	3	Post shutdown (new controls)	0.546
V2	N	April 4 - 5, 2020	3	Post shutdown (new controls)	0.0371
V2	N	April 7 - 8, 2020	3	Post shutdown (new controls)	0.318
V2	N	April 10 - 11, 2020	3	Post shutdown (new controls)	0.16
V2	N	April 13 - 14 2020	3	Post shutdown (new controls)	0.246
V2	N	April 16 - 17, 2020	3	Post shutdown (new controls)	0.0878
V2	N	April 19 - 20, 2020	3	Post shutdown (new controls)	0.325
V2	N	April 22 - 23, 2020	3	Post shutdown (new controls)	0.133
V2	N	April 25 - 26, 2020	3	Post shutdown (new controls)	0.0512
V2	N	April 28 - 29, 2020	3	Post shutdown (new controls)	0.168
V2	N	May 1 - 2, 2020	3	Post shutdown (new controls)	0.1
V3	N	April 4 - 5, 2020	3	Post shutdown (new controls)	0.27
V3	N	April 7 - 8, 2020	3	Post shutdown (new controls)	0.198
V3	N	April 10 - 11, 2020	3	Post shutdown (new controls)	0.198
V3	N	April 13 - 14 2020	3	Post shutdown (new controls)	0.65
V3	N	April 16 - 17, 2020	3	Post shutdown (new controls)	0.0918
V3	N	April 22 - 23, 2020	3	Post shutdown (new controls)	0.137
V3	N	April 28 - 29, 2020	3	Post shutdown (new controls)	0.16
V3	N	May 1 - 2, 2020	3	Post shutdown (new controls)	0.239
V4	N	April 4 - 5, 2020	3	Post shutdown (new controls)	0.125
V4	N	April 7 - 8, 2020	3	Post shutdown (new controls)	0.266
V4	N	April 10 - 11, 2020	3	Post shutdown (new controls)	0.0393
V4	N	April 13 - 14 2020	3	Post shutdown (new controls)	0.0361

V4	N	April 16 - 17, 2020	3	Post shutdown (new controls)	0.313
V4	N	April 19 - 20, 2020	3	Post shutdown (new controls)	0.12
V4	N	April 22 - 23, 2020	3	Post shutdown (new controls)	0.135
V4	N	April 25 - 26, 2020	3	Post shutdown (new controls)	0.14
V4	N	May 1 - 2, 2020	3	Post shutdown (new controls)	0.253
V5	N	April 7 - 8, 2020	3	Post shutdown (new controls)	0.175
V5	N	April 10 - 11, 2020	3	Post shutdown (new controls)	0.0614
V5	N	April 13 - 14 2020	3	Post shutdown (new controls)	0.119
V5	N	April 16 - 17, 2020	3	Post shutdown (new controls)	0.0603
V5	N	April 19 - 20, 2020	3	Post shutdown (new controls)	0.123
V5	N	April 22 - 23, 2020	3	Post shutdown (new controls)	0.305
V5	N	April 25 - 26, 2020	3	Post shutdown (new controls)	0.046
V5	N	April 28 - 29, 2020	3	Post shutdown (new controls)	0.191
V5	N	May 1 - 2, 2020	3	Post shutdown (new controls)	0.288

Terumo (CO)

Site	Background	Date	Time	Conc_ppb	LOD_ppb
1	N	August 24 - 25, 2018	Pre control	1.010	
1	N	August 25 - 26, 2018	Pre control	0.394	
1	N	August 26 - 27, 2018	Pre control	0.557	
1	N	August 27 - 28, 2018	Pre control	0.170	
1	N	August 28 - 29, 2018	Pre control	0.280	
1	N	August 29 - 30, 2018	Pre control	0.622	
1	N	August 30 - 31, 2018	Pre control	0.263	
2	N	August 24 - 25, 2018	Pre control	3.570	
2	N	August 25 - 26, 2018	Pre control	0.855	
2	N	August 26 - 27, 2018	Pre control	1.820	
2	N	August 27 - 28, 2018	Pre control	0.269	
2	N	August 28 - 29, 2018	Pre control	0.887	
2	N	August 29 - 30, 2018	Pre control	3.090	
2	N	August 30 - 31, 2018	Pre control	1.150	
3	N	August 24 - 25, 2018	Pre control	2.390	
3	N	August 25 - 26, 2018	Pre control	1.490	
3	N	August 26 - 27, 2018	Pre control	1.870	
3	N	August 27 - 28, 2018	Pre control	2.510	
3	N	August 28 - 29, 2018	Pre control	1.270	
3	N	August 29 - 30, 2018	Pre control	0.965	
3	N	August 30 - 31, 2018	Pre control	1.520	
4	N	August 24 - 25, 2018	Pre control	0.677	
4	N	August 25 - 26, 2018	Pre control	0.564	
4	N	August 26 - 27, 2018	Pre control	0.497	
4	N	August 27 - 28, 2018	Pre control	1.060	
4	N	August 28 - 29, 2018	Pre control	0.758	
4	N	August 29 - 30, 2018	Pre control	0.670	
4	N	August 30 - 31, 2018	Pre control	0.784	
NREL	Y	September 9 - 10, 2018	Pre control	ND	0.0453
La Casa	Y	September 9 - 10, 2018	Pre control	0.167	
1	N	October 17 - 18, 2018	Post control	ND	0.0453
1	N	October 18 - 19, 2018	Post control	0.125	
1	N	October 19 - 20, 2018	Post control	0.240	
1	N	October 20 - 21, 2018	Post control	0.452	
1	N	October 21 - 22, 2018	Post control	0.236	
1	N	October 22 - 23, 2018	Post control	0.231	
1	N	October 23 - 24, 2018	Post control	0.326	
2	N	October 17 - 18, 2018	Post control	0.257	
2	N	October 18 - 19, 2018	Post control	0.188	
2	N	October 19 - 20, 2018	Post control	0.682	
2	N	October 20 - 21, 2018	Post control	0.709	
2	N	October 21 - 22, 2018	Post control	0.542	
2	N	October 22 - 23, 2018	Post control	0.186	
2	N	October 23 - 24, 2018	Post control	0.444	
3	N	October 17 - 18, 2018	Post control	0.198	
3	N	October 18 - 19, 2018	Post control	1.120	

3	N	October 19 - 20, 2018	Post control	0.686	
3	N	October 20 - 21, 2018	Post control	0.663	
3	N	October 21 - 22, 2018	Post control	0.329	
3	N	October 22 - 23, 2018	Post control	0.417	
3	N	October 23 - 24, 2018	Post control	0.446	
4	N	October 17 - 18, 2018	Post control	0.197	
4	N	October 18 - 19, 2018	Post control	0.513	
4	N	October 19 - 20, 2018	Post control	ND	0.0453
4	N	October 20 - 21, 2018	Post control	0.287	
4	N	October 21 - 22, 2018	Post control	0.198	
4	N	October 22 - 23, 2018	Post control	0.228	
4	N	October 23 - 24, 2018	Post control	0.525	
5	N	October 17 - 18, 2018	Post control	0.225	
5	N	October 18 - 19, 2018	Post control	ND	0.0453
5	N	October 19 - 20, 2018	Post control	ND	0.0453
5	N	October 20 - 21, 2018	Post control	0.240	
5	N	October 21 - 22, 2018	Post control	0.245	
5	N	October 22 - 23, 2018	Post control	ND	0.0453
5	N	October 23 - 24, 2018	Post control	0.199	
6	N	October 17 - 18, 2018	Post control	ND	0.0453
6	N	October 18 - 19, 2018	Post control	0.204	
6	N	October 19 - 20, 2018	Post control	0.175	
6	N	October 20 - 21, 2018	Post control	0.259	
6	N	October 21 - 22, 2018	Post control	0.208	
6	N	October 22 - 23, 2018	Post control	0.305	
6	N	October 23 - 24, 2018	Post control	0.508	
7	N	October 17 - 18, 2018	Post control	ND	0.0453
7	N	October 18 - 19, 2018	Post control	0.148	
7	N	October 19 - 20, 2018	Post control	0.201	
7	N	October 20 - 21, 2018	Post control	0.269	
7	N	October 21 - 22, 2018	Post control	0.258	
7	N	October 22 - 23, 2018	Post control	ND	0.0453
7	N	October 23 - 24, 2018	Post control	0.382	
8	N	October 17 - 18, 2018	Post control	ND	0.0453
8	N	October 18 - 19, 2018	Post control	0.088	
8	N	October 19 - 20, 2018	Post control	0.330	
8	N	October 20 - 21, 2018	Post control	0.318	
8	N	October 21 - 22, 2018	Post control	ND	0.0453
8	N	October 22 - 23, 2018	Post control	ND	0.0453
8	N	October 23 - 24, 2018	Post control	0.476	
Welch	Y	October 26 - 27, 2018	Post control	0.200	
Welch	Y	October 27 - 28, 2018	Post control	ND	0.0453
Welch	Y	October 28 - 29, 2018	Post control	ND	0.0453
Welch	Y	October 29 - 30, 2018	Post control	ND	0.0453
NREL	Y	October 26 - 27, 2018	Post control	0.345	
NREL	Y	October 27 - 28, 2018	Post control	0.580	
NREL	Y	October 28 - 29, 2018	Post control	0.114	

Terumo (CO)

NREL	Y	October 29 - 30, 2018	Post control	0.169	
Arvada	Y	October 26 - 27, 2018	Post control	0.259	
Arvada	Y	October 27 - 28, 2018	Post control	0.374	
Arvada	Y	October 28 - 29, 2018	Post control	ND	0.0453
Arvada	Y	October 29 - 30, 2018	Post control	ND	0.0453
La Casa	Y	October 26 - 27, 2018	Post control	ND	0.0453
La Casa	Y	October 27 - 28, 2018	Post control	ND	0.0453
La Casa	Y	October 28 - 29, 2018	Post control	ND	0.0453
La Casa	Y	October 29 - 30, 2018	Post control	ND	0.0453

Viant (MI)

Site	Background	Date	Conc_ppb	LOD_ppb
1	N	November 28 - 29, 2018	0.4722	
1	N	November 28 - 29, 2019	0.4833	
1	N	March 27 - 28, 2019	0.1500	
1	N	July 9 - 10, 2019	0.5444	
1	N	August 12 - 13, 2019	0.1167	
1	N	September 10 - 11, 2019	0.2222	
1	N	October 16 - 17, 2019	0.1889	
1	N	November 19 - 20, 2019	0.0611	
1	N	December 12 - 13, 2019	0.0556	
2	N	March 27 - 28, 2019	1.1500	
3	N	March 27 - 28, 2019	ND	0.0617
4	N	March 27 - 28, 2019	0.0861	
5	N	March 27 - 28, 2019	0.1590	
5	N	March 27 - 28, 2019	0.1580	
6	N	March 27 - 28, 2019	0.0712	
7	N	March 27 - 28, 2019	0.1660	
8	N	March 27 - 28, 2019	0.0683	
8	N	March 27 - 28, 2019	ND	0.0617
9	N	March 27 - 28, 2019	0.2030	
10	Y	March 27 - 28, 2019	0.1040	
11	Y	March 27 - 28, 2019	0.0978	
12	Y	March 27 - 28, 2019	0.1180	
13	N	March 27 - 28, 2019	0.1920	
14	N	March 27 - 28, 2019	0.1900	
15	Y	March 27 - 28, 2019	0.1020	
16	N	March 27 - 28, 2019	0.1160	

Sterigenics (IL)

Site	Background	Date	Day_Night	Conc_ppb	LOD_ppb
Willowbrook Village Hall	N	11/16/2018		0.458	
Willowbrook Village Hall	N	11/19/2018		3.394	
Willowbrook Village Hall	N	11/19/2018		3.506	
Willowbrook Village Hall	N	11/23/2018		0.158	
Willowbrook Village Hall	N	11/25/2018		2.278	
Willowbrook Village Hall	N	11/28/2018		1.017	
Willowbrook Village Hall	N	12/1/2018		0.933	
Willowbrook Village Hall	N	12/1/2018		1.056	
Willowbrook Village Hall	N	12/6/2018		2.994	
Willowbrook Village Hall	N	12/7/2018		0.409	
Willowbrook Village Hall	N	12/7/2018		0.457	
Willowbrook Village Hall	N	12/10/2018		0.167	
Willowbrook Village Hall	N	12/13/2018		1.133	
Willowbrook Village Hall	N	12/13/2018		1.183	
Willowbrook Village Hall	N	12/16/2018		0.484	
Willowbrook Village Hall	N	12/19/2018		0.289	
Willowbrook Village Hall	N	12/19/2018		0.188	
Willowbrook Village Hall	N	12/22/2018		0.545	
Willowbrook Village Hall	N	12/26/2018		6.000	
Willowbrook Village Hall	N	12/26/2018		5.833	
Willowbrook Village Hall	N	12/28/2018		0.373	
Willowbrook Village Hall	N	1/2/2019		0.139	
Willowbrook Village Hall	N	1/3/2019		0.207	
Willowbrook Village Hall	N	1/3/2019		0.143	
Willowbrook Village Hall	N	1/6/2019		4.217	
Willowbrook Village Hall	N	1/6/2019		3.678	
Willowbrook Village Hall	N	1/9/2019		2.117	
Willowbrook Village Hall	N	1/12/2019		0.872	
Willowbrook Village Hall	N	1/12/2019		0.917	
Willowbrook Village Hall	N	1/15/2019		0.373	
Willowbrook Village Hall	N	1/17/2019		0.287	
Willowbrook Village Hall	N	1/17/2019		0.328	
Willowbrook Village Hall	N	1/22/2019		0.839	
Willowbrook Village Hall	N	1/24/2019		0.146	
Willowbrook Village Hall	N	1/24/2019		0.088	
Willowbrook Village Hall	N	1/27/2019		10.722	
Willowbrook Village Hall	N	2/1/2019		0.530	
Willowbrook Village Hall	N	2/1/2019		0.490	
Willowbrook Village Hall	N	2/2/2019		0.213	
Willowbrook Village Hall	N	2/5/2019		9.611	
Willowbrook Village Hall	N	2/5/2019		8.667	
Willowbrook Village Hall	N	2/8/2019		0.403	
Willowbrook Village Hall	N	2/11/2019		2.211	
Willowbrook Village Hall	N	2/11/2019		2.622	
Willowbrook Village Hall	N	2/14/2019		0.099	
EPA Willowbrook Warehouse	N	11/13/2018		1.317	

EPA Willowbrook Warehouse	N	11/16/2018	1.006	
EPA Willowbrook Warehouse	N	11/16/2018	1.006	
EPA Willowbrook Warehouse	N	11/19/2018	3.678	
EPA Willowbrook Warehouse	N	11/23/2018	0.100	
EPA Willowbrook Warehouse	N	11/28/2018	0.138	
EPA Willowbrook Warehouse	N	11/28/2018	0.633	
EPA Willowbrook Warehouse	N	12/1/2018	0.253	
EPA Willowbrook Warehouse	N	12/6/2018	6.500	
EPA Willowbrook Warehouse	N	12/6/2018	5.833	
EPA Willowbrook Warehouse	N	12/7/2018	1.256	
EPA Willowbrook Warehouse	N	12/10/2018	0.149	
EPA Willowbrook Warehouse	N	12/10/2018	0.224	
EPA Willowbrook Warehouse	N	12/13/2018	0.242	
EPA Willowbrook Warehouse	N	12/16/2018	1.172	
EPA Willowbrook Warehouse	N	12/16/2018	1.217	
EPA Willowbrook Warehouse	N	12/19/2018	0.192	
EPA Willowbrook Warehouse	N	12/22/2018	1.717	
EPA Willowbrook Warehouse	N	12/22/2018	1.428	
EPA Willowbrook Warehouse	N	12/28/2018	0.789	
EPA Willowbrook Warehouse	N	12/28/2018	0.661	
EPA Willowbrook Warehouse	N	1/2/2019	0.132	
EPA Willowbrook Warehouse	N	1/2/2019	0.220	
EPA Willowbrook Warehouse	N	1/3/2019	ND	0.045
EPA Willowbrook Warehouse	N	1/6/2019	ND	0.045
EPA Willowbrook Warehouse	N	1/9/2019	0.381	
EPA Willowbrook Warehouse	N	1/12/2019	ND	0.045
EPA Willowbrook Warehouse	N	1/15/2019	7.889	
EPA Willowbrook Warehouse	N	1/15/2019	7.944	
EPA Willowbrook Warehouse	N	1/17/2019	7.278	
EPA Willowbrook Warehouse	N	1/22/2019	2.278	
EPA Willowbrook Warehouse	N	1/22/2019	2.250	
EPA Willowbrook Warehouse	N	1/24/2019	0.156	
EPA Willowbrook Warehouse	N	1/27/2019	0.617	
EPA Willowbrook Warehouse	N	1/27/2019	0.700	
EPA Willowbrook Warehouse	N	2/1/2019	0.074	
EPA Willowbrook Warehouse	N	2/2/2019	0.127	
EPA Willowbrook Warehouse	N	2/2/2019	0.139	
EPA Willowbrook Warehouse	N	2/5/2019	14.667	
EPA Willowbrook Warehouse	N	2/8/2019	2.800	
EPA Willowbrook Warehouse	N	2/8/2019	2.389	
EPA Willowbrook Warehouse	N	2/11/2019	ND	0.045
EPA Willowbrook Warehouse	N	2/14/2019	0.414	
EPA Willowbrook Warehouse	N	2/14/2019	0.338	
Grower Middle School	N	11/19/2018	0.086	
Grower Middle School	N	11/23/2018	0.109	
Grower Middle School	N	11/25/2018	0.200	
Grower Middle School	N	11/28/2018	0.364	

Grower Middle School	N	12/1/2018	0.078	
Grower Middle School	N	12/6/2018	0.336	
Grower Middle School	N	12/7/2018	0.062	
Grower Middle School	N	12/10/2018	ND	0.045
Grower Middle School	N	12/13/2018	0.142	
Grower Middle School	N	12/16/2018	0.329	
Grower Middle School	N	12/19/2018	0.200	
Grower Middle School	N	12/22/2018	0.290	
Grower Middle School	N	12/26/2018	ND	0.045
Grower Middle School	N	12/28/2018	0.097	
Grower Middle School	N	1/2/2019	ND	0.045
Grower Middle School	N	1/3/2019	ND	0.045
Grower Middle School	N	1/6/2019	ND	0.045
Grower Middle School	N	1/9/2019	0.197	
Grower Middle School	N	1/12/2019	ND	0.045
Grower Middle School	N	1/15/2019	0.510	
Grower Middle School	N	1/17/2019	0.922	
Grower Middle School	N	1/22/2019	0.194	
Grower Middle School	N	1/24/2019	0.043	
Grower Middle School	N	1/27/2019	0.086	
Grower Middle School	N	2/1/2019	0.056	
Grower Middle School	N	2/2/2019	0.206	
Grower Middle School	N	2/5/2019	1.828	
Grower Middle School	N	2/8/2019	0.244	
Grower Middle School	N	2/11/2019	0.063	
Grower Middle School	N	2/14/2019	0.159	
West Neighborhood	N	11/19/2018	0.069	
West Neighborhood	N	11/23/2018	0.114	
West Neighborhood	N	11/25/2018	0.145	
West Neighborhood	N	11/28/2018	ND	0.045
West Neighborhood	N	12/1/2018	0.447	
West Neighborhood	N	12/6/2018	0.141	
West Neighborhood	N	12/7/2018	ND	0.045
West Neighborhood	N	12/10/2018	0.118	
West Neighborhood	N	12/13/2018	0.589	
West Neighborhood	N	12/16/2018	0.336	
West Neighborhood	N	12/19/2018	0.109	
West Neighborhood	N	12/22/2018	0.131	
West Neighborhood	N	12/26/2018	0.650	
West Neighborhood	N	12/28/2018	ND	0.045
West Neighborhood	N	1/2/2019	ND	0.045
West Neighborhood	N	1/3/2019	ND	0.045
West Neighborhood	N	1/6/2019	0.867	
West Neighborhood	N	1/9/2019	0.064	
West Neighborhood	N	1/12/2019	0.404	
West Neighborhood	N	1/15/2019	0.066	
West Neighborhood	N	1/17/2019	0.084	

West Neighborhood	N	1/22/2019	0.594	
West Neighborhood	N	1/24/2019	0.033	
West Neighborhood	N	1/27/2019	0.917	
West Neighborhood	N	2/1/2019	0.072	
West Neighborhood	N	2/2/2019	0.089	
West Neighborhood	N	2/5/2019	2.972	
West Neighborhood	N	2/8/2019	0.153	
West Neighborhood	N	2/11/2019	0.733	
West Neighborhood	N	2/14/2019	ND	0.045
Water Tower	N	11/19/2018	0.137	
Water Tower	N	11/23/2018	0.496	
Water Tower	N	11/28/2018	0.388	
Water Tower	N	12/1/2018	ND	0.045
Water Tower	N	12/6/2018	0.216	
Water Tower	N	12/7/2018	0.152	
Water Tower	N	12/10/2018	0.138	
Water Tower	N	12/13/2018	0.117	
Water Tower	N	12/16/2018	0.297	
Water Tower	N	12/19/2018	0.928	
Water Tower	N	12/22/2018	0.245	
Water Tower	N	12/26/2018	0.084	
Water Tower	N	1/2/2019	ND	0.045
Water Tower	N	1/3/2019	ND	0.045
Water Tower	N	1/6/2019	ND	0.045
Water Tower	N	1/9/2019	ND	0.045
Water Tower	N	1/12/2019	0.171	
Water Tower	N	1/15/2019	ND	0.045
Water Tower	N	1/17/2019	0.176	
Water Tower	N	1/22/2019	6.000	
Water Tower	N	1/24/2019	0.046	
Water Tower	N	1/27/2019	0.972	
Water Tower	N	2/1/2019	5.272	
Water Tower	N	2/2/2019	4.156	
Water Tower	N	2/5/2019	0.116	
Water Tower	N	2/8/2019	0.129	
Water Tower	N	2/11/2019	ND	0.045
Water Tower	N	2/14/2019	0.275	
Willow Pond Park	N	11/19/2018	0.058	
Willow Pond Park	N	11/23/2018	0.159	
Willow Pond Park	N	11/25/2018	0.192	
Willow Pond Park	N	11/28/2018	0.253	
Willow Pond Park	N	12/1/2018	0.117	
Willow Pond Park	N	12/6/2018	ND	0.045
Willow Pond Park	N	12/7/2018	0.224	
Willow Pond Park	N	12/10/2018	ND	0.045
Willow Pond Park	N	12/13/2018	0.203	
Willow Pond Park	N	12/16/2018	0.186	

Willow Pond Park	N	12/19/2018	0.303	
Willow Pond Park	N	12/22/2018	0.064	
Willow Pond Park	N	12/26/2018	0.092	
Willow Pond Park	N	12/28/2018	ND	0.045
Willow Pond Park	N	1/2/2019	0.121	
Willow Pond Park	N	1/3/2019	ND	0.045
Willow Pond Park	N	1/6/2019	ND	0.045
Willow Pond Park	N	1/9/2019	0.122	
Willow Pond Park	N	1/12/2019	ND	0.045
Willow Pond Park	N	1/15/2019	0.059	
Willow Pond Park	N	1/17/2019	0.080	
Willow Pond Park	N	1/22/2019	1.228	
Willow Pond Park	N	1/24/2019	0.063	
Willow Pond Park	N	1/27/2019	0.452	
Willow Pond Park	N	2/1/2019	2.061	
Willow Pond Park	N	2/2/2019	0.778	
Willow Pond Park	N	2/5/2019	0.097	
Willow Pond Park	N	2/8/2019	0.118	
Willow Pond Park	N	2/11/2019	0.049	
Willow Pond Park	N	2/14/2019	0.136	
Hinsdale South High School	N	11/19/2018	0.141	
Hinsdale South High School	N	11/25/2018	0.369	
Hinsdale South High School	N	11/28/2018	0.209	
Hinsdale South High School	N	12/1/2018	0.349	
Hinsdale South High School	N	12/6/2018	0.270	
Hinsdale South High School	N	12/7/2018	ND	0.045
Hinsdale South High School	N	12/10/2018	0.118	
Hinsdale South High School	N	12/13/2018	0.136	
Hinsdale South High School	N	12/16/2018	0.284	
Hinsdale South High School	N	12/19/2018	0.148	
Hinsdale South High School	N	12/22/2018	0.209	
Hinsdale South High School	N	12/26/2018	0.314	
Hinsdale South High School	N	12/28/2018	0.147	
Hinsdale South High School	N	1/2/2019	ND	0.045
Hinsdale South High School	N	1/3/2019	0.238	
Hinsdale South High School	N	1/6/2019	0.138	
Hinsdale South High School	N	1/9/2019	0.164	
Hinsdale South High School	N	1/12/2019	0.147	
Hinsdale South High School	N	1/15/2019	0.133	
Hinsdale South High School	N	1/17/2019	0.074	
Hinsdale South High School	N	1/22/2019	0.194	
Hinsdale South High School	N	1/24/2019	ND	0.045
Hinsdale South High School	N	1/27/2019	1.828	
Hinsdale South High School	N	2/1/2019	0.179	
Hinsdale South High School	N	2/2/2019	0.073	
Hinsdale South High School	N	2/5/2019	0.132	
Hinsdale South High School	N	2/8/2019	0.193	

Hinsdale South High School	N	2/11/2019		0.172	
Hinsdale South High School	N	2/14/2019		0.143	
Grower Elementary School	N	11/19/2018		0.091	
Grower Elementary School	N	11/23/2018		0.112	
Grower Elementary School	N	11/25/2018		0.228	
Grower Elementary School	N	11/28/2018		0.263	
Grower Elementary School	N	12/1/2018		0.258	
Grower Elementary School	N	12/6/2018		ND	0.045
Grower Elementary School	N	12/7/2018		0.091	
Grower Elementary School	N	12/10/2018		0.077	
Grower Elementary School	N	12/13/2018		0.223	
Grower Elementary School	N	12/16/2018		0.407	
Grower Elementary School	N	12/19/2018		0.173	
Grower Elementary School	N	12/22/2018		0.200	
Grower Elementary School	N	12/26/2018		0.276	
Grower Elementary School	N	12/28/2018		0.074	
Grower Elementary School	N	1/2/2019		0.117	
Grower Elementary School	N	1/3/2019		0.352	
Grower Elementary School	N	1/6/2019		0.138	
Grower Elementary School	N	1/12/2019		0.132	
Grower Elementary School	N	1/15/2019		ND	0.045
Grower Elementary School	N	1/17/2019		ND	0.045
Grower Elementary School	N	1/22/2019		0.332	
Grower Elementary School	N	1/24/2019		0.053	
Grower Elementary School	N	1/27/2019		0.163	
Grower Elementary School	N	2/1/2019		0.087	
Grower Elementary School	N	2/2/2019		0.119	
Grower Elementary School	N	2/5/2019		0.767	
Grower Elementary School	N	2/8/2019		0.112	
Grower Elementary School	N	2/11/2019		0.221	
Grower Elementary School	N	2/14/2019		ND	0.045
12-hr-1	Y	10/17/2018	Night	0.094	
12-hr-1	Y	11/13/2018	Day	0.261	
12-hr-2	Y	10/18/2018	Day	0.228	
12-hr-2	Y	11/16/2018	Day	0.067	
12-hr-3	Y	10/17/2018	Day	0.106	
12-hr-3	Y	11/16/2018	Day	0.083	
12-hr-4	Y	10/17/2018	Day	0.194	
12-hr-4	Y	10/17/2018	Night	0.089	
12-hr-4	Y	10/17/2018	Night	0.094	
12-hr-5	Y	10/17/2018	Night	0.233	
12-hr-5	Y	11/16/2018	Day	0.100	
12-hr-6	Y	10/18/2018	Day	0.111	
12-hr-6	Y	11/16/2018	Day	0.083	
12-hr-7	Y	10/18/2018	Day	0.117	
12-hr-7	Y	10/17/2018	Night	0.106	
12-hr-8	Y	10/18/2018	Day	0.078	

12-hr-8	Y	10/18/2018	Night	0.117
12-hr-9	Y	10/18/2018	Day	0.100
12-hr-9	Y	10/17/2018	Night	0.100
12-hr-10	Y	10/17/2018	Night	0.061
12-hr-10	Y	11/12/2018	Night	0.122
12-hr-10	Y	11/12/2018	Day	0.111
12-hr-11	Y	10/23/2018	Day	0.611
12-hr-11	Y	10/23/2018	Night	0.361
12-hr-12	Y	11/12/2018	Day	0.128
12-hr-12	Y	11/12/2018	Night	0.172
12-hr-13	Y	11/13/2018	Day	0.261
12-hr-13	Y	11/13/2018	Day	0.272
12-hr-14	Y	11/16/2018	Day	0.100
12-hr-14	Y	11/19/2018	Night	0.117

Location	Site	Background	Date	Conc_ppb	LOD_ppb
Covington County (Beckton Dickinson)	C1	N	10/3/2019	0.600	
Covington County (Beckton Dickinson)	C1	N	10/6/2019	0.078	
Covington County (Beckton Dickinson)	C1	N	10/12/2019	0.889	
Covington County (Beckton Dickinson)	C1	N	10/27/2019	1.422	
Covington County (Beckton Dickinson)	C1	N	10/30/2019	0.194	
Covington County (Beckton Dickinson)	C1	N	11/1/2019	0.094	
Covington County (Beckton Dickinson)	C1	N	11/3/2019	0.111	
Covington County (Beckton Dickinson)	C1	N	11/5/2019	0.061	
Covington County (Beckton Dickinson)	C1	N	11/8/2019	0.228	
Covington County (Beckton Dickinson)	C1	N	11/15/2019	0.367	
Covington County (Beckton Dickinson)	C2	N	10/3/2019	0.333	
Covington County (Beckton Dickinson)	C2	N	10/6/2019	0.089	
Covington County (Beckton Dickinson)	C2	N	10/18/2019	0.261	
Covington County (Beckton Dickinson)	C2	N	10/27/2019	0.178	
Covington County (Beckton Dickinson)	C2	N	10/30/2019	0.183	
Covington County (Beckton Dickinson)	C2	N	11/1/2019	0.122	
Covington County (Beckton Dickinson)	C2	N	11/3/2019	0.133	
Covington County (Beckton Dickinson)	C2	N	11/5/2019	0.094	
Covington County (Beckton Dickinson)	C2	N	11/8/2019	0.028	
Covington County (Beckton Dickinson)	C2	N	11/13/2019	0.094	
Covington County (Beckton Dickinson)	C2	N	11/15/2019	0.139	
Covington County (Beckton Dickinson)	C2	N	11/20/2019	0.561	
Covington County (Beckton Dickinson)	C2	N	11/23/2019	0.233	
Covington County (Beckton Dickinson)	C2	N	11/29/2019	0.244	
Covington County (Beckton Dickinson)	C2	N	12/5/2019	0.422	
Covington County (Beckton Dickinson)	C2	N	12/8/2019	0.106	
Covington County (Beckton Dickinson)	C2	N	12/11/2019	0.028	
Covington County (Beckton Dickinson)	C2	N	12/14/2019	0.222	
Covington County (Beckton Dickinson)	C2	N	12/17/2019	0.117	
Covington County (Beckton Dickinson)	C2	N	12/19/2019	0.117	
Covington County (Beckton Dickinson)	C2	N	12/31/2019	0.161	
Covington County (Beckton Dickinson)	C2	N	1/7/2020	0.272	
Covington County (Beckton Dickinson)	C2	N	1/10/2020	0.206	
Covington County (Beckton Dickinson)	C2	N	1/19/2020	0.078	
Covington County (Beckton Dickinson)	C2	N	1/16/2020	0.333	
Covington County (Beckton Dickinson)	C2	N	1/22/2020	0.239	
Covington County (Beckton Dickinson)	C2	N	1/25/2020	0.467	
Covington County (Beckton Dickinson)	C2	N	1/28/2020	0.561	
Covington County (Beckton Dickinson)	C2	N	2/3/2020	0.183	
Covington County (Beckton Dickinson)	C2	N	2/9/2020	0.389	
Covington County (Beckton Dickinson)	C2	N	2/15/2020	0.083	
Covington County (Beckton Dickinson)	C2	N	2/21/2020	0.267	
Covington County (Beckton Dickinson)	C2	N	2/27/2020	0.228	
Covington County (Beckton Dickinson)	C2	N	3/4/2020	0.178	
Covington County (Beckton Dickinson)	C2	N	3/10/2020	0.306	
Covington County (Beckton Dickinson)	C2	N	3/16/2020	0.050	
Covington County (Beckton Dickinson)	C2	N	3/22/2020	0.200	
Covington County (Beckton Dickinson)	C2	N	3/28/2020	0.294	
Covington County (Beckton Dickinson)	C2	N	4/3/2020	0.183	
Covington County (Beckton Dickinson)	C2	N	4/9/2020	0.350	
Covington County (Beckton Dickinson)	C2	N	4/15/2020	0.411	
Covington County (Beckton Dickinson)	C2	N	4/21/2020	0.728	
Covington County (Beckton Dickinson)	C2	N	4/27/2020	0.544	
Covington County (Beckton Dickinson)	C2	N	5/3/2020	0.372	
Covington County (Beckton Dickinson)	C2	N	5/9/2020	0.161	
Covington County (Beckton Dickinson)	C2	N	5/21/2020	0.244	

Covington County (Beckton Dickinson)	C2	N	5/27/2020	0.278
Covington County (Beckton Dickinson)	C2	N	6/2/2020	0.239
Covington County (Beckton Dickinson)	C2	N	6/14/2020	0.283
Covington County (Beckton Dickinson)	C2	N	6/20/2020	0.578
Covington County (Beckton Dickinson)	C2	N	6/26/2020	0.633
Covington County (Beckton Dickinson)	C2	N	7/2/2020	0.150
Covington County (Beckton Dickinson)	C2	N	7/8/2020	0.456
Covington County (Beckton Dickinson)	C2	N	7/14/2020	0.400
Covington County (Beckton Dickinson)	C2	N	7/20/2020	0.161
Covington County (Beckton Dickinson)	C2	N	7/26/2020	7.667
Covington County (Beckton Dickinson)	C2	N	8/1/2020	0.111
Covington County (Beckton Dickinson)	C2	N	8/7/2020	0.106
Covington County (Beckton Dickinson)	C3	N	10/3/2019	0.272
Covington County (Beckton Dickinson)	C3	N	10/6/2019	0.094
Covington County (Beckton Dickinson)	C3	N	10/12/2019	0.328
Covington County (Beckton Dickinson)	C3	N	10/18/2019	0.317
Covington County (Beckton Dickinson)	C3	N	10/24/2019	0.033
Covington County (Beckton Dickinson)	C3	N	10/27/2019	0.200
Covington County (Beckton Dickinson)	C3	N	10/30/2019	0.194
Covington County (Beckton Dickinson)	C3	N	11/1/2019	0.072
Covington County (Beckton Dickinson)	C3	N	11/3/2019	0.122
Covington County (Beckton Dickinson)	C3	N	11/8/2019	0.206
Covington County (Beckton Dickinson)	C3	N	11/13/2019	0.278
Covington County (Beckton Dickinson)	C3	N	11/15/2019	0.128
Covington County (Beckton Dickinson)	C3	N	11/20/2019	0.322
Covington County (Beckton Dickinson)	C3	N	11/23/2019	0.161
Covington County (Beckton Dickinson)	C3	N	11/29/2019	0.161
Covington County (Beckton Dickinson)	C3	N	12/5/2019	0.267
Covington County (Beckton Dickinson)	C3	N	12/8/2019	0.100
Covington County (Beckton Dickinson)	C3	N	12/11/2019	0.100
Covington County (Beckton Dickinson)	C3	N	12/14/2019	0.128
Covington County (Beckton Dickinson)	C3	N	12/17/2019	0.211
Covington County (Beckton Dickinson)	C3	N	12/19/2019	0.367
Covington County (Beckton Dickinson)	C3	N	12/31/2019	0.050
Covington County (Beckton Dickinson)	C3	N	1/4/2020	0.067
Covington County (Beckton Dickinson)	C3	N	1/7/2020	0.272
Covington County (Beckton Dickinson)	C3	N	1/10/2020	0.050
Covington County (Beckton Dickinson)	C3	N	1/14/2020	0.261
Covington County (Beckton Dickinson)	C3	N	1/19/2020	0.261
Covington County (Beckton Dickinson)	C3	N	1/22/2020	0.222
Covington County (Beckton Dickinson)	C3	N	1/25/2020	0.283
Covington County (Beckton Dickinson)	C3	N	1/28/2020	0.089
Covington County (Beckton Dickinson)	C3	N	1/30/2020	0.094
Covington County (Beckton Dickinson)	C3	N	2/3/2020	0.044
Covington County (Beckton Dickinson)	C3	N	2/9/2020	0.300
Covington County (Beckton Dickinson)	C3	N	2/15/2020	0.161
Covington County (Beckton Dickinson)	C3	N	2/21/2020	0.272
Covington County (Beckton Dickinson)	C3	N	2/27/2020	0.100
Covington County (Beckton Dickinson)	C3	N	3/4/2020	0.289
Covington County (Beckton Dickinson)	C3	N	3/10/2020	0.050
Covington County (Beckton Dickinson)	C3	N	3/16/2020	0.367
Covington County (Beckton Dickinson)	C3	N	3/22/2020	0.117
Covington County (Beckton Dickinson)	C3	N	3/28/2020	0.061
Covington County (Beckton Dickinson)	C3	N	4/3/2020	0.161
Covington County (Beckton Dickinson)	C3	N	4/9/2020	0.089
Covington County (Beckton Dickinson)	C3	N	4/15/2020	0.222
Covington County (Beckton Dickinson)	C3	N	4/21/2020	0.083

Covington County (Beckton Dickinson)	C3	N	4/27/2020	0.250	
Covington County (Beckton Dickinson)	C3	N	5/3/2020	0.156	
Covington County (Beckton Dickinson)	C3	N	5/9/2020	0.133	
Covington County (Beckton Dickinson)	C3	N	5/15/2020	0.472	
Covington County (Beckton Dickinson)	C3	N	5/21/2020	0.083	
Covington County (Beckton Dickinson)	C3	N	5/27/2020	0.222	
Covington County (Beckton Dickinson)	C3	N	6/2/2020	0.278	
Covington County (Beckton Dickinson)	C3	N	6/8/2020	0.317	
Covington County (Beckton Dickinson)	C3	N	6/14/2020	0.278	
Covington County (Beckton Dickinson)	C3	N	6/20/2020	0.594	
Covington County (Beckton Dickinson)	C3	N	6/26/2020	0.572	
Covington County (Beckton Dickinson)	C3	N	7/2/2020	0.150	
Covington County (Beckton Dickinson)	C3	N	7/8/2020	0.417	
Covington County (Beckton Dickinson)	C3	N	7/14/2020	0.289	
Covington County (Beckton Dickinson)	C3	N	7/20/2020	0.606	
Covington County (Beckton Dickinson)	C3	N	7/26/2020	0.294	
Covington County (Beckton Dickinson)	C3	N	8/1/2020	0.194	
Covington County (Beckton Dickinson)	C3	N	8/7/2020	0.217	
Covington County (Beckton Dickinson)	C4	N	10/3/2019	1.044	
Covington County (Beckton Dickinson)	C4	N	10/6/2019	0.917	
Covington County (Beckton Dickinson)	C4	N	10/12/2019	ND	0.250
Covington County (Beckton Dickinson)	C4	N	10/18/2019	0.422	
Covington County (Beckton Dickinson)	C4	N	10/24/2019	1.211	
Covington County (Beckton Dickinson)	C4	N	10/27/2019	0.106	
Covington County (Beckton Dickinson)	C4	N	10/30/2019	0.183	
Covington County (Beckton Dickinson)	C4	N	10/30/2019	0.094	
Covington County (Beckton Dickinson)	C4	N	11/1/2019	0.033	
Covington County (Beckton Dickinson)	C4	N	11/3/2019	0.100	
Covington County (Beckton Dickinson)	C4	N	11/5/2019	0.100	
Covington County (Beckton Dickinson)	C4	N	11/8/2019	0.094	
Covington County (Beckton Dickinson)	C4	N	11/15/2019	0.083	
Covington County (Beckton Dickinson)	C4	N	11/20/2019	0.556	
Covington County (Beckton Dickinson)	C4	N	11/23/2019	0.267	
Covington County (Beckton Dickinson)	C4	N	12/5/2019	0.506	
Covington County (Beckton Dickinson)	C4	N	12/8/2019	0.306	
Covington County (Beckton Dickinson)	C4	N	12/11/2019	0.172	
Covington County (Beckton Dickinson)	C4	N	12/11/2019	0.094	
Covington County (Beckton Dickinson)	C4	N	12/14/2019	0.111	
Covington County (Beckton Dickinson)	C4	N	12/17/2019	0.106	
Covington County (Beckton Dickinson)	C4	N	12/19/2019	0.156	
Covington County (Beckton Dickinson)	C4	N	12/31/2019	0.072	
Covington County (Beckton Dickinson)	C4	N	1/4/2020	0.139	
Covington County (Beckton Dickinson)	C4	N	1/7/2020	0.100	
Covington County (Beckton Dickinson)	C4	N	1/10/2020	0.367	
Covington County (Beckton Dickinson)	C4	N	1/19/2020	0.183	
Covington County (Beckton Dickinson)	C4	N	1/22/2020	0.433	
Covington County (Beckton Dickinson)	C4	N	1/22/2020	0.561	
Covington County (Beckton Dickinson)	C4	N	1/28/2020	0.306	
Covington County (Beckton Dickinson)	C4	N	1/30/2020	0.339	
Covington County (Beckton Dickinson)	C4	N	2/3/2020	0.094	
Covington County (Beckton Dickinson)	C4	N	2/9/2020	0.533	
Covington County (Beckton Dickinson)	C4	N	2/15/2020	0.472	
Covington County (Beckton Dickinson)	C4	N	2/15/2020	0.456	
Covington County (Beckton Dickinson)	C4	N	2/21/2020	0.394	
Covington County (Beckton Dickinson)	C4	N	2/27/2020	0.089	
Covington County (Beckton Dickinson)	C4	N	3/4/2020	0.361	
Covington County (Beckton Dickinson)	C4	N	3/10/2020	0.217	

Covington County (Beckton Dickinson)	C4	N	3/16/2020	0.578
Covington County (Beckton Dickinson)	C4	N	3/22/2020	0.561
Covington County (Beckton Dickinson)	C4	N	3/22/2020	0.439
Covington County (Beckton Dickinson)	C4	N	3/28/2020	0.244
Covington County (Beckton Dickinson)	C4	N	4/3/2020	0.094
Covington County (Beckton Dickinson)	C4	N	4/9/2020	0.311
Covington County (Beckton Dickinson)	C4	N	4/9/2020	0.183
Covington County (Beckton Dickinson)	C4	N	4/15/2020	0.161
Covington County (Beckton Dickinson)	C4	N	4/21/2020	0.378
Covington County (Beckton Dickinson)	C4	N	4/21/2020	0.244
Covington County (Beckton Dickinson)	C4	N	4/27/2020	0.050
Covington County (Beckton Dickinson)	C4	N	5/15/2020	0.394
Covington County (Beckton Dickinson)	C4	N	5/27/2020	0.144
Covington County (Beckton Dickinson)	C4	N	6/2/2020	0.494
Covington County (Beckton Dickinson)	C4	N	6/8/2020	0.111
Covington County (Beckton Dickinson)	C4	N	6/8/2020	0.194
Covington County (Beckton Dickinson)	C4	N	6/14/2020	0.483
Covington County (Beckton Dickinson)	C4	N	6/20/2020	0.406
Covington County (Beckton Dickinson)	C4	N	6/26/2020	0.194
Covington County (Beckton Dickinson)	C4	N	7/2/2020	0.133
Covington County (Beckton Dickinson)	C4	N	7/8/2020	0.794
Covington County (Beckton Dickinson)	C4	N	7/8/2020	0.083
Covington County (Beckton Dickinson)	C4	N	7/14/2020	0.117
Covington County (Beckton Dickinson)	C4	N	7/20/2020	0.217
Covington County (Beckton Dickinson)	C4	N	7/26/2020	0.839
Covington County (Beckton Dickinson)	C4	N	8/1/2020	0.550
Covington County (Beckton Dickinson)	C4	N	8/7/2020	0.356
Covington County (Beckton Dickinson)	C4	N	8/7/2020	0.161
Covington County (Beckton Dickinson)	C4	N	8/13/2020	0.094
Covington County (Beckton Dickinson)	C5	N	10/30/2019	0.200
Covington County (Beckton Dickinson)	C5	N	11/1/2019	0.050
Covington County (Beckton Dickinson)	C5	N	11/3/2019	0.122
Covington County (Beckton Dickinson)	C5	N	11/5/2019	0.050
Covington County (Beckton Dickinson)	C5	N	11/8/2019	0.122
Covington County (Beckton Dickinson)	C5	N	11/13/2019	0.100
Covington County (Beckton Dickinson)	C5	N	11/20/2019	0.450
Covington County (Beckton Dickinson)	C5	N	11/20/2019	0.422
Covington County (Beckton Dickinson)	C5	N	11/23/2019	0.067
Covington County (Beckton Dickinson)	C5	N	11/29/2019	0.078
Covington County (Beckton Dickinson)	C5	N	12/5/2019	0.339
Covington County (Beckton Dickinson)	C5	N	12/8/2019	0.061
Covington County (Beckton Dickinson)	C5	N	12/11/2019	0.117
Covington County (Beckton Dickinson)	C5	N	12/14/2019	0.078
Covington County (Beckton Dickinson)	C5	N	12/17/2019	0.194
Covington County (Beckton Dickinson)	C5	N	12/19/2019	0.233
Covington County (Beckton Dickinson)	C5	N	12/31/2019	0.078
Covington County (Beckton Dickinson)	C5	N	1/7/2020	0.122
Covington County (Beckton Dickinson)	C5	N	1/10/2020	0.144
Covington County (Beckton Dickinson)	C5	N	1/19/2020	0.311
Covington County (Beckton Dickinson)	C5	N	1/22/2020	0.156
Covington County (Beckton Dickinson)	C5	N	1/25/2020	0.100
Covington County (Beckton Dickinson)	C5	N	1/28/2020	0.428
Covington County (Beckton Dickinson)	C5	N	1/30/2020	0.200
Covington County (Beckton Dickinson)	C5	N	2/3/2020	0.183
Covington County (Beckton Dickinson)	C5	N	2/9/2020	0.078
Covington County (Beckton Dickinson)	C5	N	2/15/2020	0.400
Covington County (Beckton Dickinson)	C5	N	2/21/2020	0.628

Covington County (Beckton Dickinson)	C5	N	2/27/2020	0.117
Covington County (Beckton Dickinson)	C5	N	3/4/2020	0.178
Covington County (Beckton Dickinson)	C5	N	3/10/2020	0.350
Covington County (Beckton Dickinson)	C5	N	3/16/2020	0.078
Covington County (Beckton Dickinson)	C5	N	3/22/2020	0.194
Covington County (Beckton Dickinson)	C5	N	3/28/2020	0.211
Covington County (Beckton Dickinson)	C5	N	4/3/2020	0.217
Covington County (Beckton Dickinson)	C5	N	4/9/2020	0.317
Covington County (Beckton Dickinson)	C5	N	4/15/2020	0.189
Covington County (Beckton Dickinson)	C5	N	4/21/2020	0.389
Covington County (Beckton Dickinson)	C5	N	4/27/2020	0.194
Covington County (Beckton Dickinson)	C5	N	5/3/2020	0.200
Covington County (Beckton Dickinson)	C5	N	5/9/2020	0.456
Covington County (Beckton Dickinson)	C5	N	5/15/2020	0.167
Covington County (Beckton Dickinson)	C5	N	5/21/2020	0.389
Covington County (Beckton Dickinson)	C5	N	5/27/2020	0.300
Covington County (Beckton Dickinson)	C5	N	6/2/2020	0.317
Covington County (Beckton Dickinson)	C5	N	6/8/2020	0.078
Covington County (Beckton Dickinson)	C5	N	6/14/2020	0.344
Covington County (Beckton Dickinson)	C5	N	6/20/2020	0.283
Covington County (Beckton Dickinson)	C5	N	6/26/2020	0.683
Covington County (Beckton Dickinson)	C5	N	7/2/2020	0.361
Covington County (Beckton Dickinson)	C5	N	7/8/2020	0.628
Covington County (Beckton Dickinson)	C5	N	7/14/2020	0.300
Covington County (Beckton Dickinson)	C5	N	7/20/2020	0.189
Covington County (Beckton Dickinson)	C5	N	7/26/2020	0.356
Covington County (Beckton Dickinson)	C5	N	8/1/2020	0.672
Covington County (Beckton Dickinson)	C5	N	8/7/2020	0.656
Covington County (Beckton Dickinson)	C7	N	10/30/2019	0.089
Covington County (Beckton Dickinson)	C7	N	11/1/2019	0.033
Covington County (Beckton Dickinson)	C7	N	11/3/2019	0.194
Covington County (Beckton Dickinson)	C7	N	11/5/2019	0.111
Covington County (Beckton Dickinson)	C7	N	11/8/2019	0.122
Covington County (Beckton Dickinson)	C7	N	11/15/2019	0.067
Covington County (Beckton Dickinson)	C7	N	2/27/2020	0.156
Covington County (Beckton Dickinson)	C7	N	3/28/2020	0.333
Covington County (Beckton Dickinson)	C7	N	4/27/2020	0.483
Covington County (Beckton Dickinson)	C7	N	5/27/2020	0.206
Covington County (Beckton Dickinson)	C7	N	6/20/2020	0.928
Covington County (Beckton Dickinson)	C7	N	7/20/2020	0.572
Covington County (Beckton Dickinson)	C8	N	10/30/2019	0.122
Covington County (Beckton Dickinson)	C8	N	11/1/2019	0.056
Covington County (Beckton Dickinson)	C8	N	11/3/2019	0.122
Covington County (Beckton Dickinson)	C8	N	11/5/2019	0.122
Covington County (Beckton Dickinson)	C8	N	11/8/2019	0.044
Covington County (Beckton Dickinson)	C8	N	11/15/2019	0.044
Covington County (Beckton Dickinson)	C9	N	10/30/2019	0.133
Covington County (Beckton Dickinson)	C9	N	11/3/2019	0.144
Covington County (Beckton Dickinson)	C9	N	11/5/2019	0.100
Covington County (Beckton Dickinson)	C9	N	11/8/2019	0.033
Covington County (Beckton Dickinson)	C9	N	11/15/2019	0.244
Fulton County (Serlization Services of Georgia)	F1	N	1/16/2020	0.094
Fulton County (Serlization Services of Georgia)	F1	N	1/16/2020	0.094
Fulton County (Serlization Services of Georgia)	F1	N	1/22/2020	0.161
Fulton County (Serlization Services of Georgia)	F1	N	1/28/2020	0.456
Fulton County (Serlization Services of Georgia)	F1	N	2/3/2020	0.194
Fulton County (Serlization Services of Georgia)	F1	N	2/9/2020	0.244

Fulton County (Serlization Services of Georgia)	F1	N	2/15/2020	0.217
Fulton County (Serlization Services of Georgia)	F1	N	2/21/2020	0.356
Fulton County (Serlization Services of Georgia)	F1	N	3/4/2020	0.478
Fulton County (Serlization Services of Georgia)	F1	N	3/16/2020	0.206
Fulton County (Serlization Services of Georgia)	F1	N	3/22/2020	0.172
Fulton County (Serlization Services of Georgia)	F1	N	4/3/2020	0.900
Fulton County (Serlization Services of Georgia)	F1	N	4/9/2020	0.500
Fulton County (Serlization Services of Georgia)	F1	N	4/15/2020	0.328
Fulton County (Serlization Services of Georgia)	F1	N	4/21/2020	0.356
Fulton County (Serlization Services of Georgia)	F1	N	4/27/2020	0.833
Fulton County (Serlization Services of Georgia)	F1	N	5/9/2020	0.722
Fulton County (Serlization Services of Georgia)	F1	N	5/15/2020	0.317
Fulton County (Serlization Services of Georgia)	F1	N	5/21/2020	0.611
Fulton County (Serlization Services of Georgia)	F1	N	5/27/2020	0.344
Fulton County (Serlization Services of Georgia)	F1	N	6/2/2020	0.222
Fulton County (Serlization Services of Georgia)	F1	N	6/8/2020	0.633
Fulton County (Serlization Services of Georgia)	F1	N	6/14/2020	0.161
Fulton County (Serlization Services of Georgia)	F1	N	6/20/2020	0.267
Fulton County (Serlization Services of Georgia)	F1	N	6/26/2020	0.650
Fulton County (Serlization Services of Georgia)	F1	N	7/2/2020	0.661
Fulton County (Serlization Services of Georgia)	F1	N	7/8/2020	0.217
Fulton County (Serlization Services of Georgia)	F1	N	7/14/2020	0.794
Fulton County (Serlization Services of Georgia)	F1	N	7/20/2020	0.400
Fulton County (Serlization Services of Georgia)	F1	N	7/26/2020	0.872
Fulton County (Serlization Services of Georgia)	F1	N	8/1/2020	0.433
Fulton County (Serlization Services of Georgia)	F1	N	8/7/2020	1.206
Fulton County (Serlization Services of Georgia)	F1	N	8/13/2020	0.889
Fulton County (Serlization Services of Georgia)	F2	N	1/28/2020	1.594
Fulton County (Serlization Services of Georgia)	F2	N	2/3/2020	0.311
Fulton County (Serlization Services of Georgia)	F2	N	2/9/2020	0.211
Fulton County (Serlization Services of Georgia)	F2	N	2/15/2020	0.628
Fulton County (Serlization Services of Georgia)	F2	N	2/21/2020	1.556
Fulton County (Serlization Services of Georgia)	F2	N	2/27/2020	0.572
Fulton County (Serlization Services of Georgia)	F2	N	3/4/2020	1.572
Fulton County (Serlization Services of Georgia)	F2	N	3/10/2020	0.456
Fulton County (Serlization Services of Georgia)	F2	N	3/16/2020	0.444
Fulton County (Serlization Services of Georgia)	F2	N	3/22/2020	0.533
Fulton County (Serlization Services of Georgia)	F2	N	3/28/2020	0.383
Fulton County (Serlization Services of Georgia)	F2	N	4/3/2020	1.311
Fulton County (Serlization Services of Georgia)	F2	N	4/9/2020	0.722
Fulton County (Serlization Services of Georgia)	F2	N	4/15/2020	1.522
Fulton County (Serlization Services of Georgia)	F2	N	4/21/2020	0.461
Fulton County (Serlization Services of Georgia)	F2	N	4/27/2020	2.006
Fulton County (Serlization Services of Georgia)	F2	N	4/27/2020	1.933
Fulton County (Serlization Services of Georgia)	F2	N	5/9/2020	1.272
Fulton County (Serlization Services of Georgia)	F2	N	5/15/2020	0.250
Fulton County (Serlization Services of Georgia)	F2	N	5/21/2020	0.322
Fulton County (Serlization Services of Georgia)	F2	N	5/27/2020	0.183
Fulton County (Serlization Services of Georgia)	F2	N	6/2/2020	0.528
Fulton County (Serlization Services of Georgia)	F2	N	6/8/2020	0.461
Fulton County (Serlization Services of Georgia)	F2	N	6/8/2020	0.450
Fulton County (Serlization Services of Georgia)	F2	N	6/20/2020	0.494
Fulton County (Serlization Services of Georgia)	F2	N	6/26/2020	0.956
Fulton County (Serlization Services of Georgia)	F2	N	7/2/2020	0.961
Fulton County (Serlization Services of Georgia)	F2	N	7/8/2020	0.206
Fulton County (Serlization Services of Georgia)	F2	N	7/8/2020	0.256
Fulton County (Serlization Services of Georgia)	F2	N	7/14/2020	0.289

Fulton County (Serlization Services of Georgia)	F2	N	7/20/2020	0.256
Fulton County (Serlization Services of Georgia)	F2	N	7/26/2020	0.478
Fulton County (Serlization Services of Georgia)	F2	N	8/1/2020	0.106
Fulton County (Serlization Services of Georgia)	F2	N	8/13/2020	0.278
Fulton County (Serlization Services of Georgia)	F2	N	8/13/2020	0.378
Fulton County (Serlization Services of Georgia)	F3	N	8/13/2020	0.978
General Coffee State Park	General Coffee State Park	Y	9/19/2019	0.344
General Coffee State Park	General Coffee State Park	Y	10/12/2019	0.033
General Coffee State Park	General Coffee State Park	Y	10/24/2019	0.039
General Coffee State Park	General Coffee State Park	Y	11/5/2019	0.094
General Coffee State Park	General Coffee State Park	Y	11/19/2019	0.067
General Coffee State Park	General Coffee State Park	Y	11/29/2019	0.083
General Coffee State Park	General Coffee State Park	Y	12/11/2019	0.150
General Coffee State Park	General Coffee State Park	Y	12/23/2019	0.022
General Coffee State Park	General Coffee State Park	Y	1/4/2020	0.200
General Coffee State Park	General Coffee State Park	Y	1/28/2020	0.194
General Coffee State Park	General Coffee State Park	Y	2/9/2020	0.044
General Coffee State Park	General Coffee State Park	Y	2/21/2020	0.044
General Coffee State Park	General Coffee State Park	Y	3/4/2020	0.261
General Coffee State Park	General Coffee State Park	Y	3/22/2020	0.078
General Coffee State Park	General Coffee State Park	Y	3/28/2020	0.067
General Coffee State Park	General Coffee State Park	Y	4/9/2020	0.094
General Coffee State Park	General Coffee State Park	Y	4/27/2020	0.594
General Coffee State Park	General Coffee State Park	Y	5/3/2020	0.350
General Coffee State Park	General Coffee State Park	Y	5/15/2020	0.317
General Coffee State Park	General Coffee State Park	Y	6/2/2020	0.111
General Coffee State Park	General Coffee State Park	Y	6/8/2020	0.267
General Coffee State Park	General Coffee State Park	Y	6/20/2020	0.289
General Coffee State Park	General Coffee State Park	Y	7/14/2020	0.844
General Coffee State Park	General Coffee State Park	Y	7/21/2020	0.211
General Coffee State Park	General Coffee State Park	Y	8/7/2020	0.189
South Dekalb	NR - 285	Y	3/10/2020	0.206
South Dekalb	NR - 285	Y	3/22/2020	0.206
South Dekalb	NR - 285	Y	5/9/2020	0.039
South Dekalb	NR - 285	Y	6/14/2020	0.144
Cobb County (Sterigenics)	S1	N	9/30/2019	0.106
Cobb County (Sterigenics)	S1	N	10/3/2019	0.172
Cobb County (Sterigenics)	S1	N	10/6/2019	0.033
Cobb County (Sterigenics)	S1	N	10/12/2019	0.117
Cobb County (Sterigenics)	S1	N	10/18/2019	0.056
Cobb County (Sterigenics)	S1	N	10/24/2019	0.056
Cobb County (Sterigenics)	S1	N	11/20/2019	0.150
Cobb County (Sterigenics)	S1	N	11/23/2019	0.200
Cobb County (Sterigenics)	S1	N	11/29/2019	0.089
Cobb County (Sterigenics)	S1	N	12/5/2019	0.089
Cobb County (Sterigenics)	S1	N	12/11/2019	0.122
Cobb County (Sterigenics)	S1	N	12/17/2019	0.206
Cobb County (Sterigenics)	S1	N	12/31/2019	0.028
Cobb County (Sterigenics)	S1	N	1/4/2020	0.133
Cobb County (Sterigenics)	S1	N	1/10/2020	0.167
Cobb County (Sterigenics)	S1	N	1/16/2020	0.389
Cobb County (Sterigenics)	S1	N	1/22/2020	0.406
Cobb County (Sterigenics)	S1	N	1/28/2020	0.178
Cobb County (Sterigenics)	S1	N	2/3/2020	0.472
Cobb County (Sterigenics)	S1	N	2/9/2020	0.428
Cobb County (Sterigenics)	S1	N	2/15/2020	0.156
Cobb County (Sterigenics)	S1	N	2/21/2020	1.433

Cobb County (Sterigenics)	S1	N	2/27/2020	0.256
Cobb County (Sterigenics)	S1	N	3/4/2020	0.100
Cobb County (Sterigenics)	S1	N	3/10/2020	0.183
Cobb County (Sterigenics)	S1	N	3/28/2020	0.100
Cobb County (Sterigenics)	S1	N	4/3/2020	0.072
Cobb County (Sterigenics)	S1	N	4/9/2020	0.244
Cobb County (Sterigenics)	S1	N	4/15/2020	0.122
Cobb County (Sterigenics)	S1	N	4/21/2020	0.478
Cobb County (Sterigenics)	S1	N	4/27/2020	0.061
Cobb County (Sterigenics)	S1	N	5/3/2020	0.317
Cobb County (Sterigenics)	S1	N	5/9/2020	0.672
Cobb County (Sterigenics)	S1	N	5/15/2020	0.061
Cobb County (Sterigenics)	S1	N	5/21/2020	0.039
Cobb County (Sterigenics)	S1	N	5/27/2020	0.389
Cobb County (Sterigenics)	S1	N	6/2/2020	0.317
Cobb County (Sterigenics)	S1	N	6/8/2020	0.100
Cobb County (Sterigenics)	S1	N	6/14/2020	0.717
Cobb County (Sterigenics)	S1	N	6/20/2020	0.222
Cobb County (Sterigenics)	S1	N	6/26/2020	0.244
Cobb County (Sterigenics)	S1	N	7/2/2020	0.078
Cobb County (Sterigenics)	S1	N	7/8/2020	0.183
Cobb County (Sterigenics)	S1	N	7/14/2020	0.083
Cobb County (Sterigenics)	S1	N	7/20/2020	0.611
Cobb County (Sterigenics)	S1	N	8/1/2020	0.372
Cobb County (Sterigenics)	S1	N	8/7/2020	0.572
Cobb County (Sterigenics)	S1	N	8/13/2020	0.311
Cobb County (Sterigenics)	S2	N	9/24/2019	0.183
Cobb County (Sterigenics)	S2	N	9/26/2019	0.122
Cobb County (Sterigenics)	S2	N	9/30/2019	0.167
Cobb County (Sterigenics)	S2	N	10/3/2019	0.222
Cobb County (Sterigenics)	S2	N	10/6/2019	0.067
Cobb County (Sterigenics)	S2	N	10/12/2019	0.150
Cobb County (Sterigenics)	S2	N	10/18/2019	0.039
Cobb County (Sterigenics)	S2	N	10/24/2019	0.028
Cobb County (Sterigenics)	S2	N	11/20/2019	0.106
Cobb County (Sterigenics)	S2	N	11/23/2019	0.217
Cobb County (Sterigenics)	S2	N	11/29/2019	0.089
Cobb County (Sterigenics)	S2	N	12/5/2019	0.094
Cobb County (Sterigenics)	S2	N	12/11/2019	0.017
Cobb County (Sterigenics)	S2	N	12/17/2019	0.200
Cobb County (Sterigenics)	S2	N	1/10/2020	0.178
Cobb County (Sterigenics)	S2	N	1/16/2020	0.144
Cobb County (Sterigenics)	S2	N	1/22/2020	0.056
Cobb County (Sterigenics)	S2	N	1/28/2020	0.222
Cobb County (Sterigenics)	S2	N	2/3/2020	0.433
Cobb County (Sterigenics)	S2	N	2/15/2020	0.444
Cobb County (Sterigenics)	S2	N	2/27/2020	0.083
Cobb County (Sterigenics)	S2	N	3/4/2020	0.228
Cobb County (Sterigenics)	S2	N	3/10/2020	0.133
Cobb County (Sterigenics)	S2	N	3/28/2020	0.433
Cobb County (Sterigenics)	S2	N	4/3/2020	0.050
Cobb County (Sterigenics)	S2	N	4/9/2020	0.300
Cobb County (Sterigenics)	S2	N	4/15/2020	0.267
Cobb County (Sterigenics)	S2	N	4/21/2020	0.228
Cobb County (Sterigenics)	S2	N	4/27/2020	0.700
Cobb County (Sterigenics)	S2	N	5/15/2020	0.317
Cobb County (Sterigenics)	S2	N	5/21/2020	0.433

Cobb County (Sterigenics)	S2	N	5/27/2020	0.439
Cobb County (Sterigenics)	S2	N	6/2/2020	0.422
Cobb County (Sterigenics)	S2	N	6/8/2020	0.361
Cobb County (Sterigenics)	S2	N	6/14/2020	0.578
Cobb County (Sterigenics)	S2	N	6/20/2020	0.328
Cobb County (Sterigenics)	S2	N	6/26/2020	0.283
Cobb County (Sterigenics)	S2	N	7/2/2020	0.111
Cobb County (Sterigenics)	S2	N	7/8/2020	0.100
Cobb County (Sterigenics)	S2	N	7/14/2020	0.222
Cobb County (Sterigenics)	S2	N	7/20/2020	0.172
Cobb County (Sterigenics)	S2	N	7/26/2020	0.122
Cobb County (Sterigenics)	S2	N	8/1/2020	0.556
Cobb County (Sterigenics)	S2	N	8/7/2020	0.850
Cobb County (Sterigenics)	S2	N	8/13/2020	0.550
Cobb County (Sterigenics)	S3	N	9/24/2019	0.300
Cobb County (Sterigenics)	S3	N	9/26/2019	0.056
Cobb County (Sterigenics)	S3	N	9/30/2019	0.033
Cobb County (Sterigenics)	S3	N	10/3/2019	0.094
Cobb County (Sterigenics)	S3	N	10/6/2019	0.128
Cobb County (Sterigenics)	S3	N	10/12/2019	0.117
Cobb County (Sterigenics)	S3	N	10/18/2019	0.100
Cobb County (Sterigenics)	S3	N	10/24/2019	0.033
Cobb County (Sterigenics)	S3	N	11/20/2019	0.183
Cobb County (Sterigenics)	S3	N	11/23/2019	0.228
Cobb County (Sterigenics)	S3	N	11/29/2019	0.139
Cobb County (Sterigenics)	S3	N	12/5/2019	0.017
Cobb County (Sterigenics)	S3	N	12/11/2019	0.061
Cobb County (Sterigenics)	S3	N	12/17/2019	0.044
Cobb County (Sterigenics)	S3	N	12/31/2019	0.017
Cobb County (Sterigenics)	S3	N	1/4/2020	0.111
Cobb County (Sterigenics)	S3	N	1/10/2020	0.067
Cobb County (Sterigenics)	S3	N	1/22/2020	0.339
Cobb County (Sterigenics)	S3	N	1/28/2020	0.406
Cobb County (Sterigenics)	S3	N	2/3/2020	0.233
Cobb County (Sterigenics)	S3	N	2/9/2020	0.378
Cobb County (Sterigenics)	S3	N	2/15/2020	0.239
Cobb County (Sterigenics)	S3	N	2/21/2020	0.106
Cobb County (Sterigenics)	S3	N	2/27/2020	0.172
Cobb County (Sterigenics)	S3	N	3/4/2020	0.244
Cobb County (Sterigenics)	S3	N	3/10/2020	0.117
Cobb County (Sterigenics)	S3	N	3/28/2020	0.156
Cobb County (Sterigenics)	S3	N	4/3/2020	0.344
Cobb County (Sterigenics)	S3	N	4/9/2020	0.106
Cobb County (Sterigenics)	S3	N	4/15/2020	0.211
Cobb County (Sterigenics)	S3	N	4/21/2020	0.533
Cobb County (Sterigenics)	S3	N	4/27/2020	0.233
Cobb County (Sterigenics)	S3	N	5/3/2020	0.528
Cobb County (Sterigenics)	S3	N	5/9/2020	0.261
Cobb County (Sterigenics)	S3	N	5/15/2020	0.111
Cobb County (Sterigenics)	S3	N	5/21/2020	0.278
Cobb County (Sterigenics)	S3	N	5/27/2020	0.078
Cobb County (Sterigenics)	S3	N	6/2/2020	0.461
Cobb County (Sterigenics)	S3	N	6/8/2020	0.217
Cobb County (Sterigenics)	S3	N	6/14/2020	0.094
Cobb County (Sterigenics)	S3	N	6/20/2020	0.100
Cobb County (Sterigenics)	S3	N	6/26/2020	0.856
Cobb County (Sterigenics)	S3	N	7/2/2020	0.422

Cobb County (Sterigenics)	S3	N	7/8/2020	0.244	
Cobb County (Sterigenics)	S3	N	7/14/2020	0.150	
Cobb County (Sterigenics)	S3	N	7/20/2020	0.494	
Cobb County (Sterigenics)	S3	N	7/26/2020	0.700	
Cobb County (Sterigenics)	S3	N	8/1/2020	0.328	
Cobb County (Sterigenics)	S3	N	8/7/2020	0.828	
Cobb County (Sterigenics)	S3	N	8/13/2020	0.506	
Cobb County (Sterigenics)	S4	N	9/24/2019	0.206	
Cobb County (Sterigenics)	S4	N	9/26/2019	0.394	
Cobb County (Sterigenics)	S4	N	9/30/2019	0.139	
Cobb County (Sterigenics)	S4	N	10/3/2019	0.639	
Cobb County (Sterigenics)	S4	N	10/6/2019	0.033	
Cobb County (Sterigenics)	S4	N	10/12/2019	0.117	
Cobb County (Sterigenics)	S4	N	10/18/2019	0.083	
Cobb County (Sterigenics)	S4	N	10/24/2019	ND	0.025
Cobb County (Sterigenics)	S4	N	11/20/2019	0.083	
Cobb County (Sterigenics)	S4	N	11/20/2019	0.144	
Cobb County (Sterigenics)	S4	N	11/23/2019	0.056	
Cobb County (Sterigenics)	S4	N	11/29/2019	0.067	
Cobb County (Sterigenics)	S4	N	12/5/2019	0.089	
Cobb County (Sterigenics)	S4	N	12/11/2019	0.061	
Cobb County (Sterigenics)	S4	N	12/11/2019	0.244	
Cobb County (Sterigenics)	S4	N	12/17/2019	0.200	
Cobb County (Sterigenics)	S4	N	12/31/2019	0.122	
Cobb County (Sterigenics)	S4	N	1/4/2020	0.878	
Cobb County (Sterigenics)	S4	N	1/10/2020	0.306	
Cobb County (Sterigenics)	S4	N	1/16/2020	0.239	
Cobb County (Sterigenics)	S4	N	1/22/2020	0.089	
Cobb County (Sterigenics)	S4	N	1/22/2020	0.100	
Cobb County (Sterigenics)	S4	N	1/28/2020	0.272	
Cobb County (Sterigenics)	S4	N	2/3/2020	0.211	
Cobb County (Sterigenics)	S4	N	2/9/2020	0.189	
Cobb County (Sterigenics)	S4	N	2/15/2020	0.250	
Cobb County (Sterigenics)	S4	N	2/15/2020	0.233	
Cobb County (Sterigenics)	S4	N	2/21/2020	0.083	
Cobb County (Sterigenics)	S4	N	2/27/2020	0.183	
Cobb County (Sterigenics)	S4	N	3/4/2020	0.344	
Cobb County (Sterigenics)	S4	N	3/10/2020	0.167	
Cobb County (Sterigenics)	S4	N	3/28/2020	0.222	
Cobb County (Sterigenics)	S4	N	3/28/2020	0.200	
Cobb County (Sterigenics)	S4	N	4/3/2020	0.222	
Cobb County (Sterigenics)	S4	N	4/9/2020	0.378	
Cobb County (Sterigenics)	S4	N	4/9/2020	0.400	
Cobb County (Sterigenics)	S4	N	4/15/2020	0.150	
Cobb County (Sterigenics)	S4	N	4/21/2020	0.161	
Cobb County (Sterigenics)	S4	N	4/21/2020	0.533	
Cobb County (Sterigenics)	S4	N	4/27/2020	0.494	
Cobb County (Sterigenics)	S4	N	4/30/2020	0.644	
Cobb County (Sterigenics)	S4	N	5/9/2020	0.294	
Cobb County (Sterigenics)	S4	N	5/9/2020	0.400	
Cobb County (Sterigenics)	S4	N	5/15/2020	0.161	
Cobb County (Sterigenics)	S4	N	5/21/2020	0.144	
Cobb County (Sterigenics)	S4	N	5/27/2020	0.656	
Cobb County (Sterigenics)	S4	N	6/2/2020	0.500	
Cobb County (Sterigenics)	S4	N	6/8/2020	0.394	
Cobb County (Sterigenics)	S4	N	6/8/2020	0.111	
Cobb County (Sterigenics)	S4	N	6/14/2020	0.083	

Cobb County (Sterigenics)	S4	N	6/20/2020	0.272
Cobb County (Sterigenics)	S4	N	6/26/2020	0.433
Cobb County (Sterigenics)	S4	N	7/2/2020	0.472
Cobb County (Sterigenics)	S4	N	7/8/2020	0.244
Cobb County (Sterigenics)	S4	N	7/14/2020	0.478
Cobb County (Sterigenics)	S4	N	7/20/2020	0.250
Cobb County (Sterigenics)	S4	N	7/26/2020	0.294
Cobb County (Sterigenics)	S4	N	8/1/2020	0.461
Cobb County (Sterigenics)	S4	N	8/7/2020	0.328
Cobb County (Sterigenics)	S4	N	8/7/2020	0.483
Cobb County (Sterigenics)	S4	N	8/13/2020	0.411
Cobb County (Sterigenics)	S5	N	9/24/2019	1.133
Cobb County (Sterigenics)	S5	N	9/30/2019	0.178
Cobb County (Sterigenics)	S5	N	7/8/2020	0.333
Cobb County (Sterigenics)	S6	N	12/31/2019	0.078
Cobb County (Sterigenics)	S6	N	1/4/2020	0.139
Cobb County (Sterigenics)	S6	N	5/15/2020	0.478
Cobb County (Sterigenics)	S6	N	6/20/2020	0.133
Cobb County (Sterigenics)	S6	N	7/20/2020	0.167
Cobb County (Sterigenics)	S7	N	12/31/2019	0.094
Cobb County (Sterigenics)	S7	N	1/22/2020	0.056
Cobb County (Sterigenics)	S7	N	2/27/2020	0.083
Cobb County (Sterigenics)	S7	N	3/28/2020	0.189
Cobb County (Sterigenics)	S7	N	4/27/2020	0.228
Cobb County (Sterigenics)	S7	N	5/15/2020	0.811
Cobb County (Sterigenics)	S7	N	5/27/2020	0.606
Cobb County (Sterigenics)	S7	N	6/20/2020	0.344
Cobb County (Sterigenics)	S7	N	7/20/2020	0.378
South Dekalb	South Dekalb	Y	8/13/2019	0.056
South Dekalb	South Dekalb	Y	8/16/2019	0.061
South Dekalb	South Dekalb	Y	8/16/2019	0.111
South Dekalb	South Dekalb	Y	9/4/2019	0.056
South Dekalb	South Dekalb	Y	9/19/2020	0.050
South Dekalb	South Dekalb	Y	9/20/2019	0.089
South Dekalb	South Dekalb	Y	9/24/2019	0.194
South Dekalb	South Dekalb	Y	9/26/2019	0.183
South Dekalb	South Dekalb	Y	9/30/2019	0.133
South Dekalb	South Dekalb	Y	10/3/2019	0.161
South Dekalb	South Dekalb	Y	10/6/2019	0.078
South Dekalb	South Dekalb	Y	10/12/2019	0.183
South Dekalb	South Dekalb	Y	10/19/2019	0.056
South Dekalb	South Dekalb	Y	10/30/2019	0.072
South Dekalb	South Dekalb	Y	11/8/2019	0.078
South Dekalb	South Dekalb	Y	11/13/2019	0.072
South Dekalb	South Dekalb	Y	11/15/2019	0.144
South Dekalb	South Dekalb	Y	11/20/2019	0.417
South Dekalb	South Dekalb	Y	11/23/2019	0.044
South Dekalb	South Dekalb	Y	11/29/2019	0.139
South Dekalb	South Dekalb	Y	12/5/2019	0.089
South Dekalb	South Dekalb	Y	12/8/2019	0.028
South Dekalb	South Dekalb	Y	12/11/2019	0.017
South Dekalb	South Dekalb	Y	12/14/2019	0.122
South Dekalb	South Dekalb	Y	12/17/2019	0.083
South Dekalb	South Dekalb	Y	12/19/2019	0.156
South Dekalb	South Dekalb	Y	12/31/2019	0.072
South Dekalb	South Dekalb	Y	1/4/2020	0.089
South Dekalb	South Dekalb	Y	1/7/2020	0.022

South Dekalb	South Dekalb	Y	1/6/2020	0.133
South Dekalb	South Dekalb	Y	1/22/2020	0.233
South Dekalb	South Dekalb	Y	1/28/2020	0.256
South Dekalb	South Dekalb	Y	2/3/2020	0.178
South Dekalb	South Dekalb	Y	2/3/2020	0.150
South Dekalb	South Dekalb	Y	2/15/2020	0.261
South Dekalb	South Dekalb	Y	2/21/2020	0.094
South Dekalb	South Dekalb	Y	2/27/2020	0.072
South Dekalb	South Dekalb	Y	3/4/2020	0.117
South Dekalb	South Dekalb	Y	3/4/2020	0.478
South Dekalb	South Dekalb	Y	3/10/2020	0.133
South Dekalb	South Dekalb	Y	3/16/2020	0.256
South Dekalb	South Dekalb	Y	3/22/2020	0.083
South Dekalb	South Dekalb	Y	3/22/2020	0.167
South Dekalb	South Dekalb	Y	3/28/2020	0.206
South Dekalb	South Dekalb	Y	4/3/2020	0.556
South Dekalb	South Dekalb	Y	4/3/2020	0.261
South Dekalb	South Dekalb	Y	4/9/2020	0.083
South Dekalb	South Dekalb	Y	4/15/2020	0.089
South Dekalb	South Dekalb	Y	4/21/2020	0.467
South Dekalb	South Dekalb	Y	4/27/2020	0.511
South Dekalb	South Dekalb	Y	5/3/2020	0.222
South Dekalb	South Dekalb	Y	5/3/2020	0.333
South Dekalb	South Dekalb	Y	5/9/2020	0.089
South Dekalb	South Dekalb	Y	5/15/2020	0.278
South Dekalb	South Dekalb	Y	5/21/2020	0.111
South Dekalb	South Dekalb	Y	6/2/2020	0.294
South Dekalb	South Dekalb	Y	6/2/2020	0.078
South Dekalb	South Dekalb	Y	6/8/2020	0.639
South Dekalb	South Dekalb	Y	6/14/2020	0.217
South Dekalb	South Dekalb	Y	6/20/2020	0.456
South Dekalb	South Dekalb	Y	6/26/2020	0.333
South Dekalb	South Dekalb	Y	7/2/2020	0.444
South Dekalb	South Dekalb	Y	7/2/2020	0.517
South Dekalb	South Dekalb	Y	7/8/2020	0.450
South Dekalb	South Dekalb	Y	7/14/2020	0.594
South Dekalb	South Dekalb	Y	7/20/2020	0.022
